# Crystal structures of BMPRII extracellular domain in binary and ternary receptor complexes with BMP10

**DOI:** 10.1038/s41467-022-30111-2

**Published:** 2022-05-03

**Authors:** Jingxu Guo, Bin Liu, Midory Thorikay, Minmin Yu, Xiaoyan Li, Zhen Tong, Richard M. Salmon, Randy J. Read, Peter ten Dijke, Nicholas W. Morrell, Wei Li

**Affiliations:** 1 Department of Medicine, University of Cambridge School of Clinical Medicine, Cambridge, CB2 0QQ United Kingdom; 2 Department of Cell and Chemical Biology and Oncode Institute, Leiden University Medical Centre, Leiden, The Netherlands; 3 MRC Laboratory of Molecular Biology, Francis Crick Avenue, Cambridge, CB2 0QH United Kingdom; 4 Cambridge Institute for Medical Research, The Keith Peters Building, Cambridge Biomedical Campus, Hills Road, Cambridge, CB2 0XY United Kingdom

**Keywords:** Transforming growth factor beta, Growth factor signalling, Vascular diseases, X-ray crystallography

## Abstract

Heterozygous mutations in *BMPR2* (bone morphogenetic protein (BMP) receptor type II) cause pulmonary arterial hypertension. BMPRII is a receptor for over 15 BMP ligands, but why *BMPR2* mutations cause lung-specific pathology is unknown. To elucidate the molecular basis of BMP:BMPRII interactions, we report crystal structures of binary and ternary BMPRII receptor complexes with BMP10, which contain an ensemble of seven different BMP10:BMPRII 1:1 complexes. BMPRII binds BMP10 at the knuckle epitope, with the A-loop and β4 strand making BMPRII-specific interactions. The BMPRII binding surface on BMP10 is dynamic, and the affinity is weaker in the ternary complex than in the binary complex. Hydrophobic core and A-loop interactions are important in BMPRII-mediated signalling. Our data reveal how BMPRII is a low affinity receptor, implying that forming a signalling complex requires high concentrations of BMPRII, hence mutations will impact on tissues with highest *BMPR2* expression such as the lung vasculature.

## Abstract (web summary)

Mutations in BMPR2 is the major genetic cause for pulmonary arterial hypertension (PAH). Here by solving crystal structures of BMPRII in binary and ternary receptor complexes with BMP10, the authors report the molecular recognition between BMPRII and BMP10, and its implication in PAH.

## Introduction

Transforming growth factor β (TGF-β) family cytokines control many fundamental biological processes, from the establishment of body patterning during embryonic development, to the maintenance of adult homeostasis in vasculature, wound repair, and immune response. The TGF-β ligands are dimers and initiate signal transduction by forming a signalling complex with two copies of a type I receptor and two copies of a type II receptor; all are single-pass transmembrane proteins containing a small extracellular ligand-binding domain (ECD) and an intracellular kinase domain (ICD). Upon signalling complex formation, the constitutively active type II receptor phosphorylates and activates the type I receptor; subsequently the type I receptor phosphorylates Smad1/5 or Smad2/3 to regulate transcriptional responses.

There are more than 30 genes encoding TGF-β ligands which can be broadly divided into three subfamilies, the TGF-βs, the activins, and the bone morphogenetic proteins (BMPs). Their signalling is mediated by only 7 type I receptors (Activin receptor-like kinase 1-7 (ALK1-7)) and 5 type II receptors (TGF-β receptor type II (TGFβRII), Activin receptor type 2 A and 2B (ActRIIA and ActRIIB), BMP receptor type II (BMPRII) and Anti-Mullerian hormone (AMH) receptor type II (AMHRII)). Although some ligand:receptor interactions are of high affinity and specificity, such as TGFβRII for TGF-β1 and TGF-β3, ALK1 for BMP9 and BMP10, many are promiscuous. For example, ActRIIA/B bind to and mediate the signalling from both activin ligands and some BMP ligands, whereas BMPRII is the low-affinity type II receptor for over 15 BMP ligands^1–3^. Although BMPRII binds BMP10 with the highest affinity among different ligands^1^, its affinity is still at least 10-fold weaker than other high-affinity cognate receptor:ligand interactions in the TGF-β family^1^, and around 10-fold weaker than ActRIIB binding to BMP10^2^. How BMPRII regulates BMP signalling through low-affinity interactions is not known.

BMPRII is unique among TGF-β family receptors because it possesses a long carboxy-terminal tail of more than 500 amino acids after the kinase domain. Although both ActRIIA/B and BMPRII can mediate signalling from different BMPs, the phenotypes of knockout mice, tissue distribution, and the effects of mutations in human diseases are very different among these three type II receptors. Loss of function mutations in *BMPR2* are the major genetic cause for pulmonary arterial hypertension (PAH)^4,5^, the consequences of which include remodelling of the pulmonary arteries, elevated right ventricular pressure, right ventricle hypertrophy and eventually heart failure. There is currently no cure for PAH and it represents a significant unmet medical need. Of note, no mutations in genes encoding ActRIIA or ActRIIB have been reported in PAH patients.

Around 668 *BMPR2* mutations have been identified in PAH cohorts to date^6^. Most mutations result in haploinsufficiency, but around 25% are missense mutations in the ECD and ICD. Some missense mutations, including many cysteine substitutions, cause protein misfolding and retention in the endoplasmic reticulum^7,8^. Other mutations in the ECD do not affect cell surface localisation and their impact on ligand binding and signalling activity remains unclear.

Why *BMPR2* mutations cause PAH despite the receptor being rather ubiquitously expressed in the body is intriguing, suggesting a pivotal role for BMPRII in the lung vasculature. Of note, BMPRII is particularly highly expressed in lung vascular endothelial cells, where it mediates the signalling from circulating BMP9 and BMP10. The unique features of these two ligands include binding with very high affinity and specificity to the endothelial-specific type I receptor ALK1, and the co-receptor endoglin (ENG). The importance of ALK1 and ENG in the vasculature is underpinned by human genetics showing that autosomal dominant loss-of-function mutations in these two genes cause Hereditary Haemorrhagic Telangiectasia (HHT)^9,10^, a vascular abnormality characterised by telangiectases (broken capillaries) in the nasal mucosa, gastrointestinal tract and skin, and larger arteriovenous malformations in brain, lungs and liver, which can be life-threatening. Mutations in *ALK1*, *ENG*, *GDF2* (encoding BMP9) and *BMP10* have also been reported in PAH patients^11^. Thus, human genetics strongly supports a role for BMPRII and BMPRII/ALK1-mediated BMP9 and 10 signalling in lung endothelial cells in the pathogenesis of PAH. In accordance with this, we have recently shown that endogenous BMP9 plays a critical role in the maintenance of endothelial integrity, particularly in the pulmonary vasculature^12^.

Despite two decades of extensive research on BMPRII since the discovery of *BMPR2* mutations in PAH, crystal structures of BMPRII signalling complexes are yet to be solved, perhaps due to the difficulty of obtaining crystals from the low affinity receptor:ligand complexes. Here we report crystal structures of BMP10:BMPRII complex in two crystal forms, and the ALK1:BMP10:BMPRII signalling complex. We show that although BMPRII utilises the same hydrophobic core as ActRIIA/B and binds to BMP10 at the knuckle epitope, the longer A-loop and finger 3 loop (F3-loop) in BMPRII make unique interactions. Importantly, we show that BMPRII makes fewer interactions with BMP10 and has lower binding affinity for BMP10 in the ternary complex than in the binary complex, suggesting a mechanism for the transient nature of the BMPRII-mediated signalling. Surprisingly, we found that BMP10 fingertip 3/4 preferentially adopts either an extended or a bent conformation in different protein-protein interaction contexts. Such conformational plasticity is also present in BMP9; which may be another unique feature of BMP9 and 10 contributing to their signalling specificity.

## Results

### Overall structures of BMP10:BMPRII and ALK1:BMP10:BMPRII complexes

**Fig. 1:**
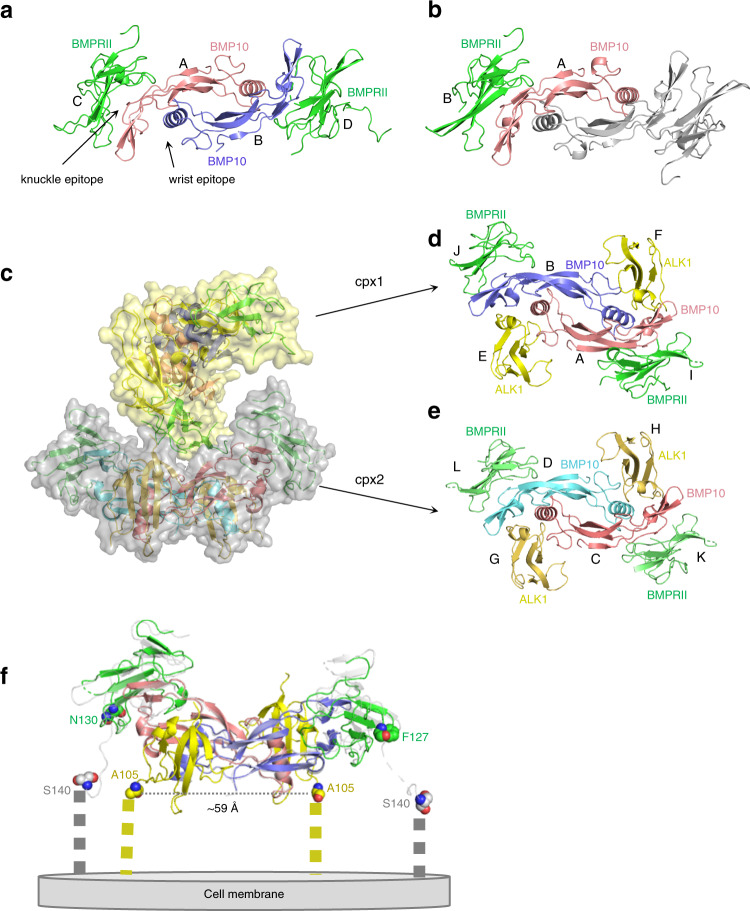
Overall structures of BMP10:BMPRII and ALK1:BMP10:BMPRII complexes. **a** One asymmetric unit of the BMP10:BMPRII 1.48 Å crystal structure. Chain identities (IDs) A to D are labelled. BMP10 is coloured in coral and light purple, BMPRII coloured in green. **b** 2.4 Å structure of BMP10:BMPRII with chain IDs labelled. Only one monomer of BMP10 (in coral) and BMPRII (in green) in an asymmetric unit. One symmetry-related molecule is shown in grey to illustrate the BMP10 dimer bound to two copies of BMPRII. **c**–**e** Overall structure of the ALK1:BMP10:BMPRII complex. Four copies of each BMP10, ALK1 and BMPRII monomers are found in one asymmetric unit, forming two copies of ternary signalling complexes shown in semi-transparent yellow and grey surface. Chain IDs in complex 1 (cpx1) (**d**) and cpx2 (**e**) are shown. In cpx1, BMP10 monomers are coloured in coral and light purple, ALK1 in yellow and BMPRII coloured in green. In cpx2, BMP10 monomers are coloured in coral and cyan, ALK1 in dark yellow, BMPRII in green. **f** An illustration of BMPRII-signalling complex in relation to cell surface. The last residues in ALK1 and BMPRII ECD cDNA-encoded sequences are 118 and 150, respectively. The last residues that can be seen in the crystal structures are shown in spheres and labelled. The 1.48 Å BMP10:BMPRII structure (in grey and semi-transparent) is superimposed on the ternary signalling complex (coloured as in Fig. 1d, cpx1) to show positions of further modelled sequence in BMPRII C-termini. The C-terminal 10–13 residues in both ALK1 and BMPRII that are not visible in the structure are represented by thick dashed lines.

We solved crystal structures of the BMPRII binary complex with BMP10 in two different crystal forms, at 1.48 Å and 2.4 Å, respectively (Supplementary Table 1 and Supplementary Fig 1). In the 1.48 Å structure, the asymmetric unit contains one BMP10 dimer with two copies of BMPRII (Fig. 1a). In the 2.4 Å structure, there is only one copy each of BMP10 and BMPRII monomers in an asymmetric unit. The dimeric receptor complex can be generated with a symmetry-related molecule (Fig. 1b). In addition, we solved the crystal structure of the ALK1:BMP10:BMPRII ternary signalling complex (Fig. 1c–e). There are 4 copies each of BMP10, ALK1 and BMPRII monomers in an asymmetric unit, assembled into two copies of BMP10 ternary signalling complexes (Fig. 1d, e). Complex 1 (cpx1) contains chains A, B, E, F, I and J (Fig. 1d), and cpx2 contains chains C, D, G, H, K and L (Fig. 1e). In the ternary complex, while good densities were observed for all BMP10 and ALK1 chains, densities for BMPRII were relatively weaker.

BMP ligands have been typically described as a hand, and the receptor binding sites designated as wrist epitopes, knuckle epitopes and fingertips (Supplementary Fig 2). Overall, the BMPRII-signalling complex assembly resembles the reported BMP family complexes, in which ALK1 binds BMP10 at the wrist epitope and BMPRII binds at the knuckle epitope. No direct contact is present between ALK1 and BMPRII (Fig. 1d, e). The position of the signalling complex in relation to cell surface is illustrated in Fig. 1f.

### Comparison of ALK1 binding sites in binary and ternary complexes

**Fig. 2:**
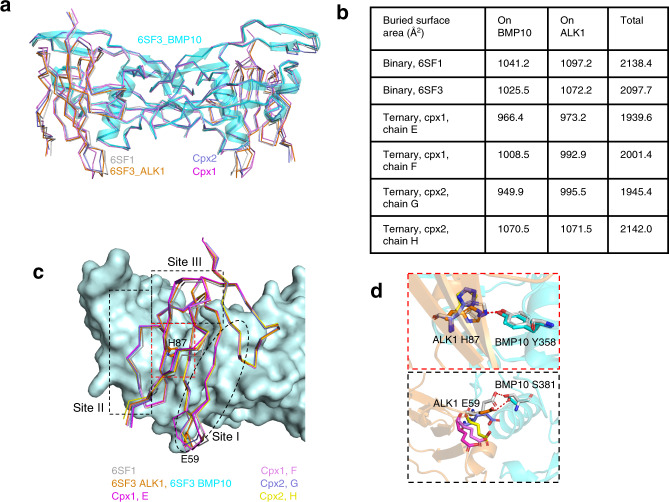
Comparison of ALK1 binding sites in binary and ternary BMPRII receptor complexes. **a** Overlay of BMP10:ALK1 from the ternary signalling complex (cpx1, magenta, cpx2, purple) to those from binary complexes (PDB code 6SF1 in grey; 6SF3 in cyan for BMP10 and orange for ALK1). The backbones of all overlaid molecules are shown in ribbon. BMP10 from 6SF3 also shown in semi-transparent cartoon. Because in both 6SF1 and 6SF3, there was only one copy of BMP10:ALK1 monomer in an asymmetric unit, the dimeric receptor complexes for 6SF1 and 6SF3 were generated with a symmetry-related molecule and the two BMP10:ALK1 interfaces in 6SF1 and 6SF3 dimer would be identical. **b** Comparison of the buried surface area at the BMP10 and ALK1 interface in binary and ternary receptor complexes. **c** Overlay of all ALK1 chains, displayed in ribbon on BMP10 surface (light cyan). Four parts of BMP10 binding sites on ALK1 identified previously^13^ are highlighted by dashed lines. The colour for each chain is shown below. **d** Zoomed-in views of ALK1 H87 and E59 interaction area. H-bond interactions are shown with dotted lines. Same colour scheme as in **c**. The only interactions can be seen are in grey (from 6SF1), and orange and cyan (from 6SF3). Detailed information and the list of the interactions can be found in Supplementary Table 2.

Overlaying the ALK1:BMP10 portion of the ternary complexes onto the previously reported ALK1:BMP10 complex showed no significant conformational change (Fig. 2a). Interestingly, among the two ternary complexes in the asymmetric unit, the four ALK1-binding sites have slightly different buried interface areas (Fig. 2b). We have shown previously that the ALK1:BMP10 interface can be broadly divided into four parts, the hydrophobic core, site I which has different interactions between BMP9 and BMP10, and sites II and III that are conserved between BMP9 and BMP10 (Fig. 2c)^13^. In the ternary complex, the interactions at sites II and III are mostly maintained (Supplementary Table 2). Two noticeable changes in the interface interactions are the loss of a hydrogen (H)-bond mediated by ALK1 H87 at the centre of the hydrophobic core in cpx2, and H-bonds mediated by ALK1 E59 at site I in both ternary complexes (Fig. 2d, Supplementary Table 2). Despite such differences, we did not observe binding affinity change between BMP10 and ALK1 in the presence or absence of BMPRII (Supplementary Fig 3).

### Highly flexible BMP10:BMPRII interactions with a hinge in BMP10

Since each copy of BMPRII only contacts one copy of BMP10 (Fig. 1a), these three crystal structures contain an ensemble of seven BMP10:BMPRII 1:1 complexes in different crystal environments, and provide a unique opportunity to investigate whether there is conformational flexibility present in the BMP10:BMPRII interactions. Using the chain identities to name the seven complexes (Supplementary Table 3), they are complexes AC and BD (Fig. 1a), complex AB (Fig. 1b) and complexes AI, BJ, CK and DL from the ternary complexes (Fig. 1d, e).

**Fig. 3:**
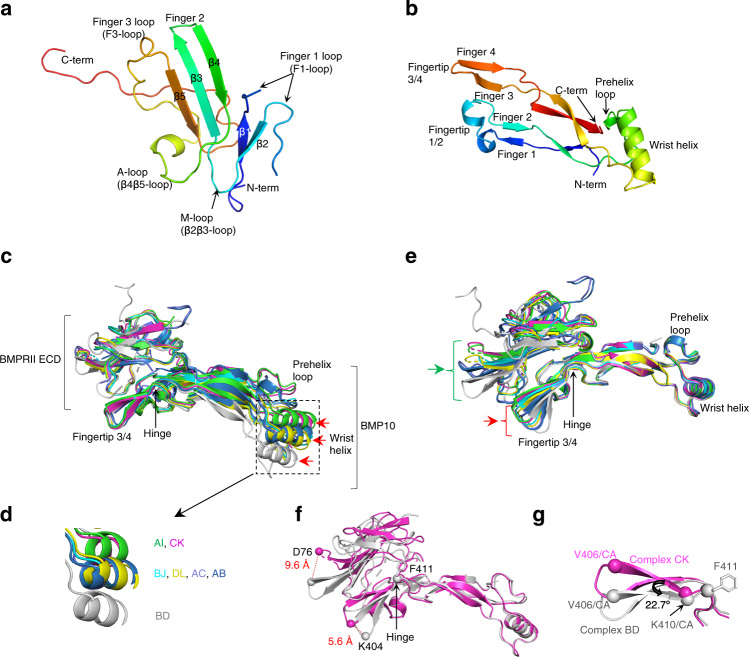
Highly flexible BMP10:BMPRII interaction with a hinge in BMP10. **a,b** Secondary structural elements of the type II receptor (**a**, represented by BMPRII) or BMP (**b**, represented by BMP10), coloured in rainbow from blue at the N-terminus to red at the C-terminus. **c**, **d** Overlay of seven BMP10:BMPRII 1:1 complexes by BMPRII (**c**) and a zoomed-in view of the wrist helix region (**d**). **e** Overlay of seven BMP10:BMPRII 1:1 complexes by BMP10 wrist helix. In **c**–**e**, complex AI in green, CK in magenta, BJ in cyan, DL in dark yellow, AC in light purple, AB in dark blue, BD in grey. In **c**, **e**, red arrows highlight the movement of BMP10 among the 7 complexes, whilst the green arrow highlights the movement of BMPRII among the 7 complexes. **f**, **g** Overlay of complexes CK (magenta) and BD (grey) by BMP10 wrist helix. **f**. The hinge region and the distances between the BMPRII finger 2 hairpins and BMP10 fingertips 3/4 are shown. **g** A zoomed-in view of the hinge area, showing the rotation angle between complex CK V406/CA, complex BD K410/CA and complex BD V406/CA is 22.7 degrees.

BMPRII has a three-finger toxin fold, with three pairs of anti-parallel beta-strands denoted as fingers 1, 2 and 3 (Fig. 3a). The A-loop connects fingers 2 and 3, and the M-loop connects fingers 1 and 2^14,15^. Each BMP monomer has been typically described as a hand with four fingers and a wrist helix (Fig. 3b)^3,16^. When overlaying seven BMP10:BMPRII 1:1 complexes by BMPRII, although the BMP10:BMPRII interface area were generally aligned, significant differences were present in the BMP10 wrist helix and prehelix loop (Fig. 3c, d). Conversely, when overlaying the seven complexes by the BMP10 wrist helix, similar shifts were seen in the BMPRII finger 2 hairpin and BMP10 fingertip 3/4 (Fig. 3e). The largest shift was between complexes CK and BD with a 5.6 Å shift in BMP10 at the fingertip 3/4, and a 9.6 Å shift in the BMPRII finger 2 hairpin (Fig. 3f and Supplementary Movie 1). A hinge region was observed in BMP10 near residue F411, where the finger 4 β-strand moved by 22.7 degrees between complex CK and complex BD (Fig. 3e–g). Of note, F411 in BMP10 and its equivalent Y416 in BMP9 are the unique insertion residues in BMP9 and BMP10 that determine the type II receptor site specificity^13^. Overall, the seven BMP10:BMPRII 1:1 complexes can be broadly arranged into three different conformations, with complexes AI and CK in one conformation that differs most from complex BD, whilst complexes BJ, DL, AC and AB are in an intermediate conformation (Fig. 3c–e).

### The binding interface between BMP10 and BMPRII

**Fig. 4:**
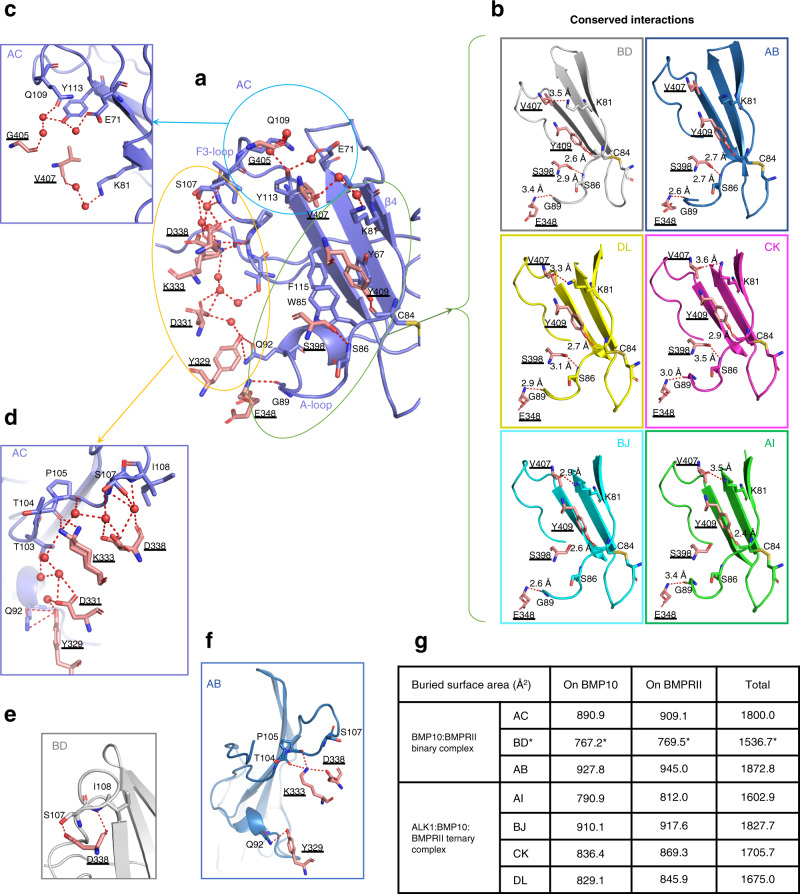
Detailed BMP10:BMPRII interface interactions. **a** BMP10:BMPRII binding interface in complex AC. BMP10 (in coral)-binding surface on BMPRII (purple) can be broadly divided into three regions. The central hydrophobic triad (Y67, W85 and F115), the β4 strand to the A-loop (green oval), the F3-loop and the region connecting the A-loop and the F3-loop (light blue circle and orange oval). **b**–**f** Detailed interactions between BMP10 and BMPRII, at the β4 strand and A-loop region in all complexes (**b**), at the F3-loop (**c**) and the regions connecting the A-loop and the F3-loop in complex AC (**d**), at BMPRII S107 region in complex BD (**e**) and AB (**f**). BMP10 is shown in coral sticks throughout **b**–**f**, whereas BMPRII is in grey for complex BD, in dark blue for complex AB, in yellow for complex DL, in magenta for complex CK, in cyan for complex BJ and in green for complex AI. In **a**–**f** red dashed lines denote H-bonds, with distance all between 2.7-3.7 Å if not labelled. Underlined residue numbers are those from BMP10, and residues numbers in normal text are those from BMPRII. **g** Buried interface area between BMP10 and BMPRII in binary and ternary receptor complexes. *Complex BD has much smaller buried surface area because some sidechains at the interface were deleted between the A-loop and F3-loop due to poor densities.

The BMP10 binding site on BMPRII consists of three areas. The central hydrophobic core, which contains the hydrophobic triad residues Y67, W85 and F115 (Fig. 4a), is conserved across the seven BMP10:BMPRII complexes. This hydrophobic triad is also present at the binding sites on ActRIIA or ActRIIB for BMP2, BMP7, BMP9, ActA and GDF11^2,15,17–22^, but not on AMHRII^16^ or TGFβRII (Supplementary Fig 4)^23–25^. The second area is the β4 strand extended to the A-loop that is unique to BMPRII (Fig. 4a, green oval). Here three H-bond interactions are formed between the mainchain atoms of C84, S86 and G89 in BMPRII, and the sidechains of Y409, S398 and the mainchain of E348 in BMP10, respectively, contacting three out of the four β-strands in the BMP10 knuckle area (Supplementary Fig 5a). Slight variations in this region among the seven complexes include the lengths of the H-bonds, missing the interaction between BMPRII S86 and BMP10 S398 in complex BJ and AI, and complexes BD, DL, CK, BJ and AI have an additional H-bond between BMPRII K81 and the mainchain of BMP10 V407 (Fig. 4b). Of note, the interaction between K81 and V407 is also present in complex AC, but instead of a direct H-bond, the interaction is mediated by two water molecules (Fig. 4c). Additional water-mediated interactions between BMP10 and BMPRII are present only in complex AC in this region (Fig. 4c). The third area is between the A-loop and the F3-loop and is different among different complexes. In complex AC, this region makes significant contact with BMP10 (Fig. 4a, orange oval), with many direct and water-mediated H-bonds (Fig. 4d). Interestingly, 5 out of 15 residues with alternate conformations in complex AC are at this area (Supplementary Table 4), suggesting that the conformation in this region is highly flexible. Consistent with this observation, in complex BD and complex AB, the F3-loop region makes different interactions with BMP10 but both involve BMP10 residue D338 and are near BMPRII residue S107 (Fig. 4 e, f). Of note, these interactions are present in all three BMP10:BMPRII 1:1 complexes from the binary complexes, but not in those from the ternary complexes. The buried surface areas at the interfaces from the ternary complexes are generally smaller than those from the binary complexes with only one exception (Fig. 4g), but in the same range as the BMP9:ActRIIB interface in the ternary signalling complexes (Supplementary Fig 5b). We questioned whether fewer interface interactions and a smaller interface area would translate to weaker BMP10:BMPRII interactions in the ternary complexes. In the surface plasmon resonance (SPR) binding assay, indeed, BMP10 bound to BMPRII-Fc surface with ~5 fold weaker affinity when ALK1 was present (Supplementary Fig 5c).

Overall, our results show that the BMP10:BMPRII interaction is highly dynamic with an ensemble of multiple conformations, and is weaker in the ternary signalling complexes compared with those in the binary complexes.

### Comparison of BMP10 binding site on BMPRII with BMP9 site on ActRIIB

**Fig. 5:**
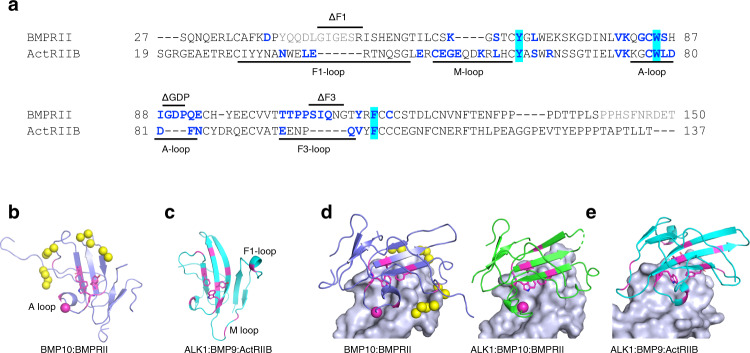
Comparison of BMP10 binding site on BMPRII with BMP9 site on ActRIIB. **a** Sequence alignment of BMPRII ECD and ActRIIB ECD. Residues at the binding interface with BMP10 or BMP9 are shown in blue. Residues that are not modelled in the BMPRII structure are shown in grey. Hydrophobic triad residues are highlighted in cyan. Positions of the four loops are highlighted below the sequence. Lines above the sequence highlight the residues deleted in the mutagenesis studies. ΔF1(F3) = deletion of finger 1 (finger 3) residues; ΔGDP = deletion of residue Gly, Asp and Pro. **b**, **c** BMP10-binding site on BMPRII (**b**, light purple) and BMP9-binding site on ActRIIB (**c**, cyan). Residues making direct interactions with BMP10 or BMP9 are coloured in magenta, with hydrophobic triad residues shown in sticks. BMP10 G89 is highlighted in a magenta sphere. Water molecules that mediate hydrogen bond interactions between BMPRII and BMP10 are shown in yellow spheres. **d** BMPRII in binary (complex AC, purple cartoon) and ternary (complex AI, green cartoon) complexes on BMP10 surface (pale blue), showing G89 (magenta spheres) docking into a deep pocket on BMP10. **e** ActRIIB (cyan cartoon) on BMP9 surface (pale blue, from PDB entry 4FAO) does not have A-loop mediated interaction. In **d**, **e**, residues making H-bond interactions shown in magenta, and hydrophobic triad shown in magenta sticks.

Sequence alignment of BMPRII and ActRIIB ECDs revealed that BMPRII has a six-residue insertion in the F1-loop, a four-residue deletion in the M-loop, a three-residue insertion in the A-loop, and a five-residue insertion in the F3-loop (Fig. 5a). Interestingly, residues from these loops are all engaged in direct interactions with BMPs (Fig. 5a). The insertions in both the A-loop and the F3-loop of BMPRII make specific interactions with BMP10 that are not present in ActRIIB (Fig. 5a, b). In contrast, the longer M-loop and the shorter F1-loop in ActRIIB make several interactions with BMP9 that are not seen in BMP10:BMPRII complexes (Fig. 5a, c)^2^. Of interest, comparing with free BMPRII structures^26^, noticeable changes can be seen in the A-loop and F3-loop (Supplementary Fig 6a), suggesting that upon binding to BMP10, these two loops need to be restrained to engage interactions.

Apart from using unique loop interactions to achieve specificity, another striking difference between ActRIIB and BMPRII is that the ActRIIB central hydrophobic core is well-shielded by surrounding H-bond interactions (Fig. 5c). In contrast, the BMPRII hydrophobic core is only partially shielded from solvent water by the conserved interactions from strand β4 and the A-loop (Fig. 5b, d). Many water-mediated H-bonds are clearly visible in complex AC from the 1.48 Å structure (Fig. 4a, c, d, 5b, d). In the BMP9:ActRIIB complex, residues surrounding the hydrophobic core make direct interactions, with two additional H-bonds from the M-loop adding further seals to the hydrophobic core (Fig. 5c, e, Supplementary Fig 6b).

### Context-dependent fingertip 3/4 conformation in BMP9 and BMP10

Since we observed three different groups of conformations in BMP10 from the seven BMP10:BMPRII 1:1 complexes (Fig. 3d), we questioned whether BMP9 and BMP10 have any preferred conformation in a defined protein-protein interaction context. To address this question, we needed as many BMP9 and BMP10 crystal structures in as many different protein interaction contexts as possible. As summarised in Supplementary Table 5, BMP9 crystal structures have been reported as free ligand form^27–29^, with prodomain^30^, with prodomain and ALK1^13^, with ALK1 and ActRIIB^2^, and with ENG^29^. For BMP10, we have previously reported its complex with ALK1^13^, and in this study with BMPRII alone, and with both ALK1 and BMPRII. Here we also solved the crystal structures of BMP10 in a non-covalent complex with its prodomain (Pro:BMP10), which provided a BMP10 conformation in yet another protein interaction context. Two crystal forms (Supplementary Table 1) were solved to 2.9 Å (crystal form 1, which contains Pro:BMP10 with mutations in the prodomain unstructured regions to promote crystal contacts), and to 3.5 Å (crystal form 2, which contains wild type (WT) Pro:BMP10), respectively (Supplementary Fig 7). The two structures overlay well in the BMP10 chains and in the prodomain regions that interact with BMP10. The prodomain regions that are not engaged in protein-protein interactions and the α5 helix are truncated or disordered, *i.e*., not visible in the crystal structures (Supplementary Fig 7, 8a). Importantly, the interface between the prodomain and BMP10 is identical in both structures and comprised of interactions from the extended β-sheet and the α2 helix (Supplementary Fig 8b), conserved with previously reported Pro:BMP9 structures^13,30^. The four BMP10 monomers in the two Pro:BMP10 structures provided the conformation of BMP10 in the prodomain-bound form for further analysis (Supplementary Fig 8c).

**Fig. 6:**
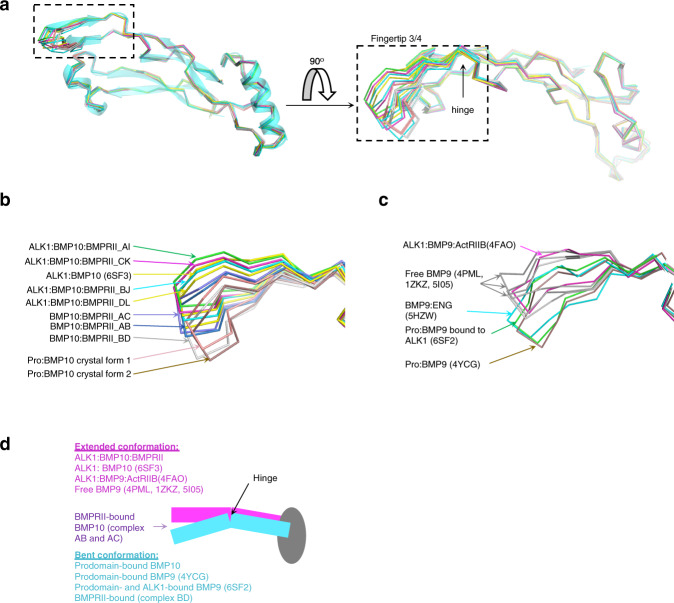
Context-dependent fingertip 3/4 conformation in BMP9 and BMP10. **a** An overlay of all BMP10 monomers from different protein interaction contexts. Sources of structures are listed in Supplementary Table 5. **b** A close-up view of fingertip 3/4 with all the structures annotated. **c** A closed-up view of fingertip 3/4 region after superposition of all BMP9 monomers from different protein interaction contexts. Sources of the structures are listed in Supplementary Table 5. The overlay of the full monomers can be found in Supplementary Fig 9. **d** A schematic diagram depicting the context-dependent conformation of fingertip 3/4 in BMP9 and BMP10.

Overlaying BMP10 monomers from seven crystal structures in four different protein interaction contexts reveals that fingertip 3/4 samples a collection of different conformations (Fig. 6a, b), starting from the hinge region (Fig. 3e, f). A similar finding was seen when overlaying BMP9 monomers from eight crystal structures in five protein interaction contexts (Supplementary Fig 9, Fig. 6c). Interestingly, annotating each BMP9 or BMP10 structure with its protein interaction context (Fig. 6b, c) revealed that in both proteins, fingertip 3/4 can adopt either an extended or a bent formation (Fig. 6d). In the presence of the prodomain, the fingertip 3/4 is found in the bent conformation for both BMP9 and BMP10. In contrast, in the free ligand form, or in the complex with ALK1 (but no prodomain), BMP9 and 10 preferentially adopt the extended conformation. ENG-bound BMP9 is closer to the prodomain-bound form, whereas BMPRII-bound BMP10 (in the absence of ALK1) mostly adopts an intermediate conformation (Fig. 6d).

Of note, both ENG and prodomain interact with BMP9 and 10 via the extended β-sheet^13,29,30^ which is not present in BMP10:BMPRII interactions. It is likely that the extended β-sheet interaction preferentially stabilizes the bent conformation in BMP9 and 10. The release of this constraint allows BMP9 and 10 to adopt the conformation required for the ternary signalling complex formation. BMPRII can bind BMP10 fingertip 3/4 region in multiple conformations thereby the affinity of the interaction might be compromised by the flexibility in this region.

### Mutagenesis studies confirming the BMP10 binding sites on BMPRII

**Fig. 7:**
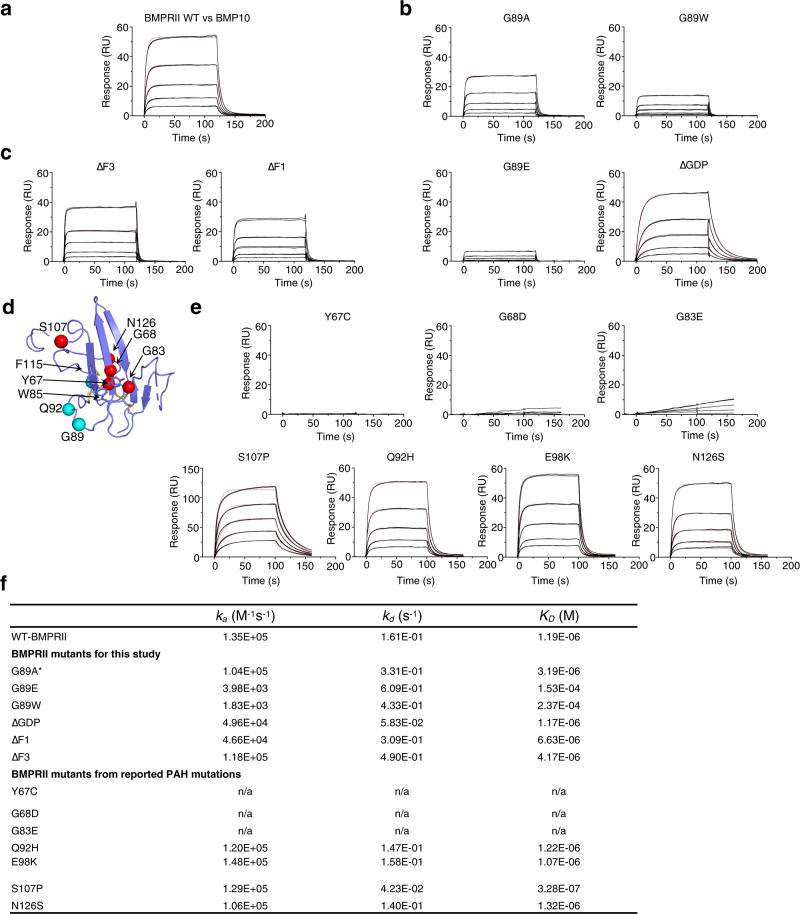
BMP10 binding to BMPRII and its mutant proteins. **a** The sensorgram of monomeric BMPRII WT ECD binding to BMP10 immobilised on a CM5 Biacore chip. Control experiments of ALK1-Fc and BMPRII-Fc binding to the same BMP10 chip are shown in Supplementary Fig 10. **b** G89-containing A-loop is essential for BMPRII binding to BMP10. **c** F1-loop and F3-loop deletion mutants binding to BMP10. **d** Locations of PAH mutations^6^ on the BMPRII ECD structure (purple cartoon). Red spheres: residues predicted to be deleterious; cyan spheres, residues predicted to be benign. **e** Sensorgrams of BMPRII ECD proteins containing PAH mutations binding to BMP10. **f**. Summary of kinetic parameters from the Biacore binding experiments. *G89A has also been found in an hereditary PAH case, but predicted to be a benign mutation. WT wild type, ECD extracellular domain, ΔF1(F3) deletion of finger 1 (finger 3) residues, ΔGDP deletion of Gly, Asp and Pro in the A-loop. M Molar concentration, s = second, *k*_*a*_ = association rate constant, *k*_*d*_ = dissociation rate constant, *K*_*D*_ = *k*_*d*_ */k*_*a*_.

Next we generated BMPRII ECD mutant proteins and evaluated whether the unique loop insertions in BMPRII (Fig. 5a) are essential for its binding to BMP10. Control SPR binding experiments show that BMP10 immobilised on the CM5 chip can bind BMPRII-Fc and ALK1-Fc with nanomolar and sub-nanomolar affinities, respectively (Supplementary Fig 10), but to monomeric BMPRII ECD with only micromolar affinity (Fig. 7a, f). Among the A-loop insertion residues, G89 docks in a deep pocket on BMP10 (Fig. 5d). We predict that a bulky residue at this position would create steric hindrance and weaken the interaction. Indeed, although BMPRII G89A binds BMP10 with less than a 2-fold reduction in affinity, G89W and G89E substitutions, which are too big to fit into the binding pocket on BMP10 (Fig. 5d), bind to BMP10 with more than 100-fold reduction in affinity, mostly due to the slower on-rates (Fig. 7b, f). The ΔGDP mutant, where three residues in the middle of the A-loop are deleted (Fig. 5a), binds to BMP10 with similar affinity to the WT, but with different kinetics (Fig. 7b, f): a slower on-rate and a slower off-rate. These data suggest that the role of the A-loop is to promote the initial anchoring on BMP10, possibly also to stabilise the interactions while BMP10 undergoes the bent-to-extended conformational change. Deleting extra residues in the finger 1 (ΔF1) and finger 3 (ΔF3) loops (Fig. 5a) resulted in 5.6- and 3.5-fold reduction in the binding affinity, respectively, suggesting both loops contribute to the interaction (Fig. 7c, f). Of note, no density for finger 1 insertion residues was observed in any of the structures, whereas F3-loops make different interactions with BMP10 only in the binary complexes.

Many non-cysteine mutations in *BMPR2* ECD were found in PAH patients (Fig. 7d), of which the mechanism for the deleterious effect is unknown. In agreement with our structures, mutations at the hydrophobic interface, such as Y67C, G68D and G83E, all led to a complete loss of BMP10 binding (Fig. 7e, f). S107 makes an additional interaction with BMP10 in binary complexes, but not in ternary complexes (Fig. 4d–f). Interestingly, S107P mutant binds to BMP10 nearly 4-fold tighter than the WT BMPRII due to a slower off-rate. Q92 contacts BMP10 in two out of three binary complexes. E98 and N126 are not at the binding interface. We did not observe any change in binding affinity for Q92H, E98K or N126S mutations (Fig. 7e, f).

### BMPRII interface residues are important for prodomain displacement

**Fig. 8:**
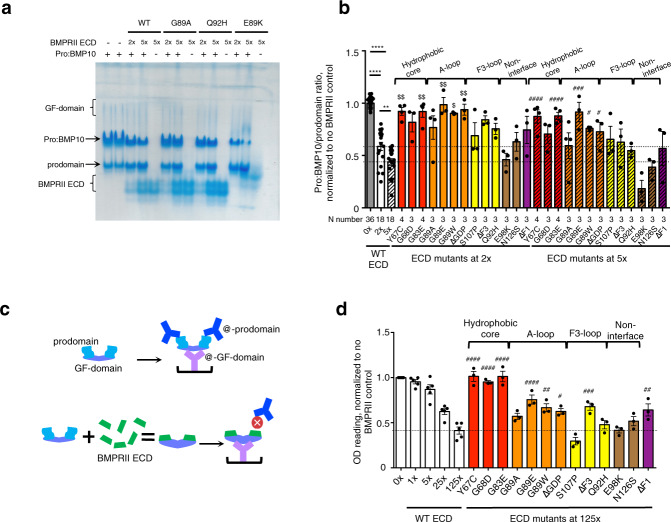
BMP10:BMPRII interaction is important for prodomain displacement. **a** A representative native PAGE of prodomain displacement experiment. **b** Quantification of native PAGE results. The band intensities of Pro:BMP10 and prodomain were obtained by densitometry using ImageJ. The ratio of Pro:BMP10/prodomain for each lane was calculated, then normalised to that of the WT control run on the same gel. Each mutant protein was run on at least three independent native PAGE experiments, with two WT controls on each gel to allow normalisation. Final N numbers for each sample are labelled below the graph. Conditions with BMPRII ECDs at 2-fold excess (2x) are shown in open bars, and those with 5x in hashed bars. The two dotted lines mark the mean values for 2x and 5x WT BMPRII, respectively. Data are presented as mean values + /− SEM. One-way ANOVA analysis for WT ECD group, ****P < 0.0001, **P < 0.01; for BMPRII at 2x excess group, Dunnett’s post test comparing with WT BMPRII ECD, ^$^*P* < 0.05, ^$$^*P* < 0.01; for BMPRII at 5x excess group, Dunnett’s post test comparing with WT BMPRII ECD, ^#^*P* < 0.05, ^###^*P* < 0.001, ^####^*P* < 0.0001. **c** Schematic depicting the experiment of the prodomain displacement by ELISA. **d** Quantitative results of prodomain displacement by ELISA. All readings are normalised to the WT on the same plate. *N* = 5 for WT ECD, *N* = 3 for each mutant protein. Each N number represents an independent ELISA experiment. Data are presented as mean values + /− SEM. One-way ANOVA analysis for BMPRII at 125x excess, with Dunnett’s post test comparing with WT BMPRII ECD (mean value shown as a dotted line). ^#^*P* < 0.05, ^##^*P* < 0.01, ^###^*P* < 0.001, ^####^*P* < 0.0001. In **b**, **d**, coloured in red are residues near the hydrophobic triad, in orange are residues from the A-loop, in yellow are F3-loop; in brown, non-interface residues, and in purple, F1-loop which is not visible in the structure. Source data, including the exact p values for **b**, **d**, are provided as a Source Data file. ECD = extracellular domain, GF-domain = growth factor domain, Pro:BMP10 = non-covalent complex of BMP10 prodomain with its GF-domain.

Since BMP10 circulates in the prodomain-bound form^31^ and the prodomain binding site overlaps with the type II receptor binding site on BMP10, the prodomain needs to be displaced for the signalling complex to form. We have previously shown that excess BMPRII ECD can displace the prodomain from Pro:BMP10^31^; here we investigate whether mutations in BMPRII would affect its prodomain displacement function. As shown previously also in Fig. 8a, on native PAGE, Pro:BMP10 alone runs as three bands, a growth factor (GF)-domain band, the Pro:BMP10 complex band and prodomain alone (Supplementary Fig 11). In the presence of WT BMPRII, there is a dose-dependent decrease of the Pro:BMP10 complex band. Different BMPRII ECD mutants showed different abilities to displace the prodomain on native PAGE. To quantify such changes, we calculated the ratio of band intensities of Pro:BMP10/prodomain to minimise the effect from loading difference (Fig. 8b). Despite some gel-to-gel variation in this assay, significant changes can be observed upon addition of 2-fold or 5-fold excess of WT BMPRII in a dose-dependent manner_._ Among the BMPRII mutant proteins, mutations in the hydrophobic cores which lost affinity for BMP10 (Fig. 7e, f) also lost the ability to displace the prodomain. Mutations in the A-loop, especially G89E, G89W and ΔGDP, significantly reduced the ability to displace the prodomain. Deletion of the F3-loop, which contains interactions only in the binary complex, reduced displacement of the prodomain. Non-interface mutations, such as E98K, N126S and ΔF1, did not interfere with the prodomain displacement function of BMPRII.

To confirm the above result, we performed a Pro:BMP10 ELISA experiment with or without pre-incubating Pro:BMP10 with BMPRII ECD. Should BMPRII displace the prodomain, we would expect to see a decreased signal in the ELISA (Fig. 8c). Control experiments showed a dose-dependent decrease of Pro:BMP10 signal with increasing concentrations of WT BMPRII ECD (Fig. 8d). We evaluated the mutant proteins in this assay along with the WT control at 125-fold excess of ECDs. Similar to the results from the native PAGE, mutations in the hydrophobic core resulted in the loss of ability to displace the prodomain; and mutants in the A-loop have reduced ability to displace the prodomain. Interestingly, deletion of the F1-loop or F3-loop also significantly reduced the ability of BMPRII to displace the prodomain.

Overall, these two independent prodomain-displacement experiments support the conclusion that mutations in the hydrophobic core and the A-loop that cause significant loss of binding affinity for BMP10 also lead to the loss of the ability to displace the prodomain.

### BMPRII interface residues are essential for the signalling activity

**Fig. 9:**
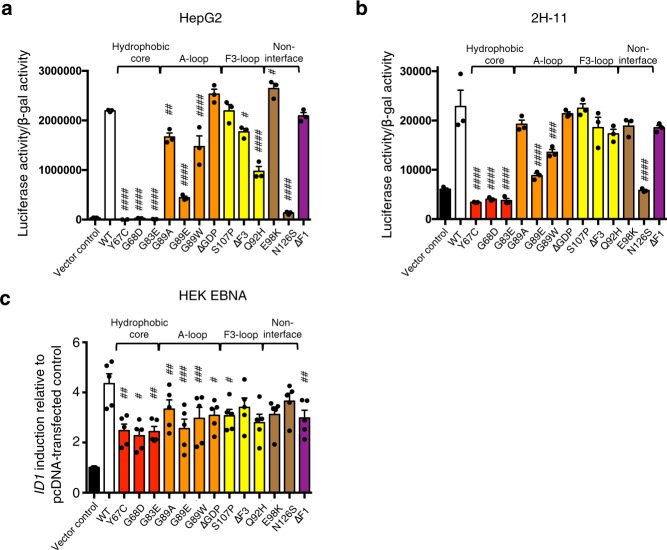
*BMPR2* mutations that disrupt BMP10 binding also result in impaired BMPRII-mediated signalling. BMPRII-mediated signalling assay was established by transfecting cells with pcDEF plasmids containing short form of BMPR2 WT or mutant sequences. **a**, **b** HepG2 (**a**) and 2H-11 (**b**) cells were seeded in 24-well plates and transfected with BRE-luc reporter, β-gal plasmid (transfection control) and mutant or WT *BMPR2* plasmids in triplicate. 48 h post-transfection, the cells were serum-starved overnight before luciferase activity was measured. **c** HEK-EBNA cells were seeded in 6 well plates and transfected with the same set of *BMPR2* plasmids over 24-hour duration before harvesting for RNA and qPCR analysis of *ID1* gene expression. Gene expression level is presented as fold-change relative to *B2M* and further normalised to control (transfected with vector only). *N* = 5, with each N number represents an independent transfection and signalling experiment. For **a**–**c**, means ± SEM are shown, data were analysed using One-way ANOVA with Dunnett’s post test for comparing with WT transfection. ^#^*P* < 0.05, ^##^*P* < 0.01, ^###^*P* < 0.001, ^####^*P* < 0.0001. Source data, including the exact p values, are provided as a Source Data file. Colour scheme and abbreviations are the same as in Fig. 8.

Finally, we investigated whether BMPRII interface residues that are important for BMP10 binding and prodomain displacement are also important in mediating BMP signalling. It is challenging to set up signalling assays using the full-length *BMPR2* gene (long form) because the expression level is very low, and transfection did not lead to an increase in BMP signalling (Supplementary Fig. 12a). However, the short form of *BMPR2* that lacks the C-terminal tail can be transfected efficiently, and the transfection alone induced Smad1/5 phosphorylation even in the absence of exogenous ligands (Supplementary Fig. 12a). This provided a unique assay to evaluate the ability of BMPRII ECD in mediating BMP signalling, which is likely in response to BMPs present in fetal bovine serum (FBS) or produced by cells in this setting. We performed BMP/Smad1/5-response element (BRE)-luciferase reporter assays by transfecting *BMPR2* short form, with or without mutations in the ECD, into HepG2 cells, an immortal human cell line from liver carcinoma, or 2H-11 cells, an immortalised mouse endothelial cell line, alongside β-Gal transfection controls (Fig. 9a, b). Comparing with WT *BMPR2* transfection, mutations in the hydrophobic core completely abolished any increase in BRE signal from *BMPR2* short form, whereas mutations such as ΔGDP, S107P, E98K or ΔF1 did not have any impact on signalling. Mutations in the A-loop that affected prodomain displacement, such as G89E and G89W, also showed reduced ability to mediate signalling. The N126S mutation completely abolished the signalling mediated by the transfected *BMPR2*. This is likely due to a folding defect because N126 is predicted to be a glycosylation site (containing N126-X-T128 consensus) by Uniprot^32^. Once it is made in a recombinant expression system, it can bind to BMP10 with similar affinity to WT BMPRII (Fig. 7e, f). Interestingly, when we performed a signalling assay by transfecting the same set of constructs into HEK EBNA cells, which is a cell line used for recombinant protein production and thus might have better folding capacity, we saw the most significantly compromised signalling for the mutations in the hydrophobic core, G89E and G89W (Fig. 9c), but not N126S. Surface biotinylation followed by ELISA quantification showed that all mutant *BMPR2* constructs expressed in HEK EBNA cells were detected at the cell surface at comparable levels (Supplementary Fig. 12b).

## Discussion

Although *BMPR2* mutations were identified as the major genetic cause for PAH in 2000, no clinically approved treatment targeting *BMPR2* has been achieved. This may be partly because it is not understood how BMPRII mediates the signalling from different BMPs and in different cell types. Although free BMPRII ECD structures were reported in 2003, a binary or ternary complex with any BMP ligand has not been reported due to the difficulty of purifying low-affinity BMPRII:ligand complexes. Here, by solving the crystal structures of BMPRII in binary and ternary complexes with BMP10, we obtained an ensemble of seven BMP10:BMPRII 1:1 complex structures which demonstrated that the specificity of BMPRII is conferred by the β4 strand and the GDP insertion in the A-loop, with the hydrophobic triad making the major contribution to binding stability. The finger 3 region interaction is highly flexible and only observed in the binary complex; and the buried surface area at the BMP10:BMPRII interface is generally smaller in the ternary complexes than in the binary complexes. Consistent with this, BMP10 binds to BMPRII with lower affinity when ALK1 is present, in contrast to the TGF-β signalling complex where binding of the low-affinity receptor ALK5 is greatly enhanced in the ternary complex when TGFβRII is present^25^.

The A-loop in BMPRII promotes the initial binding, probably to facilitate the displacement of the prodomain, also to provide an anchoring point allowing BMP10 (possibly also BMP9) to undergo the bent-to-extended conformational change in fingertip 3/4. The insertion in the A-loop in BMPRII comes at a slight cost of complex stability because the loop truncation mutant ΔGDP binds to BMP10 with both a slower on-rate and a slower off-rate.

Although BMPRII is predicted to be the type II receptor for all BMP ligands, the binding affinities were only measured in a selected set of ligands, and BMPRII also binds to ActA and Nodal with higher affinities than it binds to many BMPs^1^. Sequence alignment revealed a conserved IAP motif across all BMPRII-binding ligands (Supplementary Fig. 13). No other residues appear to be essential for interacting with BMPRII since many interactions are mediated by mainchain atoms.

Some ligands in the TGF-β family have shown a high degree of diversity in the dimer shape, such as TGF-βs and activins, where they exhibit closed and open conformations with free rotations around the centre of the dimer^33^. In contrast, BMP ligand dimers are generally rigid and display a canonical butterfly-like shape^33^. Here we show that the “wings” of the butterfly can move, adopting an extended, an intermediate, or a bent conformation depending on the protein-protein interaction context. Prodomain-binding fixes BMP9 and BMP10 in the bent conformation, presumably a more restrained conformation at least for BMP9 because the three free BMP9 structures, reported by different groups and using proteins purified by different methods, are all in the extended conformation^27–29^. Upon release of the prodomain, the fingertip 3/4 becomes more flexible; and the binding of ALK1 stabilizes it in the extended conformation. The fact that the extended conformation of BMP10 is only observed in the ternary not the BMP10:BMPRII binary complex suggests that BMP10 type II surface in the ternary complex may not be the optimum surface for BMPRII binding. This is consistent with the SPR measurement that BMPRII binds to BMP10 with lower affinity in the presence of ALK1. All these data support the dynamic and transient nature of the BMPRII-mediated ternary signalling complex. One interesting feature in the TGF-β family signalling complexes is that the ligand has high affinity for only one receptor rather than binding to both receptors with high affinity simultaneously. After the type II receptor phosphorylates the type I receptor, it is preferable for the type I receptor to dissociate from the type II receptor and bind to its own substrate such as the R-Smads to relay the signal transduction efficiently. Hence a transient and less stable ternary complex, that lasts just long enough to allow type II receptors to phosphorylate type I receptors, would be preferred over a highly stable ternary signalling complex where both type I and type II receptors bind ligands tightly. Such a complex would be most efficient in allowing the type I receptor to move on to phosphorylate its targets, possibly also to allow BMPRII to be made available for other type I receptors.

The potential drawback of such a mechanism is that high concentrations of BMPRII, or the help of a third protein such as a co-receptor, are required to stabilise the ternary signalling complex. This may provide an explanation why *BMPR2* mutations cause PAH. Among 668 *BMPR2* mutations found in PAH patients, the majority cause haploinsufficiency. Our results suggest that haploinsufficiency will have the most impact on cells and tissues with highest BMPRII expression where low-affinity BMPRII-mediated signalling is most active. Interestingly, The Human Protein Atlas shows the highest *BMPR2* mRNA levels in the lung among different tissues, and the highest in endothelial cells among the lung single cell types (Supplementary Fig. 14). Therefore, haploinsufficiency will likely have the most impact on lung endothelial cells. Since endothelial dysfunction in the pulmonary circulation is the initial trigger for PAH, this may explain why the haploinsufficiency of *BMPR2* is most likely to cause PAH despite the receptor being expressed in many tissues. Our finding supports the strategy of restoring cell surface BMPRII protein as an effective way to treat PAH.

## Methods

### Materials

All chromatography columns were from Cytiva. Crystallisation reagents and tools were purchased from Hampton Research Inc and Molecular Dimensions Ltd. DNA purification and RNA extraction kits were from Qiagen. Human Hep-G2 hepatocellular carcinoma cells (cat. No. HB-8065), mouse 2H-11 endothelial cells (cat. No. CRL-2163) and human embryonic kidney (HEK) EBNA cells (cat. No. CRL-10852) were all purchased from American Type Culture Collection (ATCC). All cell lines were cultured in Dulbecco’s modified Eagle medium (DMEM) with 10% fetal bovine serum (FBS) and 100 U/mL penicillin-streptomycin and were frequently tested for the absence of mycoplasma contamination.

### Expression and purification of Pro:BMP10

Pro:BMP10 was expressed and purified following the published method^13,31^. Briefly, cDNA of full-length open reading frame of human *BMP10* (NM_014482) was cloned into pCEP4 and transfected into HEK EBNA in DMEM supplied with 5% FBS. Human full-length *FURIN* cDNA in pCEP4 was co-transfected to facilitate processing. Serum-free chemically defined (CD) CHO (cat. No. 10743-011, Gibco) medium was applied from day 2 and conditioned media were harvested every 3–4 days for 5 harvests. To purify Pro:BMP10, conditioned medium was loaded onto 5 ml Hitrap Q columns, selected fractions containing Pro:BMP10 were concentrated and loaded onto a HiLoad Superdex 200 pg 16/600 gel filtration column. Peak fractions were further purified using a MonoP 5/50 GL column pre-equilibrated with 20 mM Tris, 50 mM NaCl, pH 7.4 and eluted with a NaCl gradient. Target fractions were concentrated and loaded onto an Superdex 200 column pre-equilibrated in 20 mM Tris, 150 mM NaCl, pH 7.4. To facilitate crystallisation, a mutant form of Pro:BMP10 was produced. Using the UCLA MBI-SERp server^34^, residues K267-E269 and E296-E297 were suggested as residues with high entropy at the protein surface, and mutating which may enhance the protein’s crystallisability via the generation of crystal contacts. Therefore these residues were all mutated to alanine using site-directed mutagenesis. The mutant Pro:BMP10 protein was expressed and purified using the same method as described for the WT Pro:BMP10.

### Expression and purification of ALK1 ECD, BMPRII ECD WT and mutants

ALK1 ECD was expressed and purified following the published method^13^. In brief, human *ACVRL1* (NM_000020, encodes ALK1) ECD cDNA (amino acids 22-118) was cloned into a pET39b vector. A TEV (Tobacco Etch Virus nuclear inclusion A endopeptidase) cleavage site was introduced at the N-terminus of ALK1 ECD. The plasmid was transformed into Rosetta DE3 bacteria and cultured in 2x YT medium at 37 °C to mid-log phase followed by IPTG (Isopropyl β-D-1-thiogalactopyranoside) induction at 22 °C overnight. The fusion protein DsbA-(His)_6_-ALK1 ECD was extracted from the periplasmic compartment according to the pET System Manual (Novagen) followed by purification using a 5 ml HisTrap Excel column (GE Healthcare). The fusion protein was incubated with a His-tagged TEV protease and dialysed in Tris-buffered saline (TBS) overnight. The cleaved DsbA-(His)_6_ and TEV were removed from ALK1 ECD using a 5 ml HisTrap Excel column. ALK1 ECD was further purified by a Superdex 75 pg 16/600 column (GE Healthcare).

Human *BMPR2* cDNA (NM_001204) encoding amino acids 27-150 was cloned into a pET39b plasmid to create a fusion WT protein DsbA-(His)_6_-BMPRII ECD. All the mutations and deletions were introduced using a Q5^®^ Site-Directed Mutagenesis Kit (New England Biolabs) following the manufacturer’s protocol and were confirmed by sequencing. The WT and mutant proteins were expressed and purified following the method described above for ALK1 ECD.

### Surface plasmon resonance analysis

Surface plasmon resonance (SPR) experiments were undertaken using the Biacore T200 system (GE Healthcare). Recombinant human BMP10 growth factor (GF)-domain (cat. No. 2926-BP-025/CF, R&D systems) was immobilised onto a Series S research grade CM5 sensor chip (cat. No. BR100530, GE Healthcare) by amine-coupling at a density of 1300 resonance units. For kinetic measurements, a series of concentrations of ALK1-Fc (cat. No. 370-AL, R&D Systems), BMPRII-Fc (cat. No. 811-BR, R&D Systems), monomeric BMPRII WT and mutant ECDs were injected in duplicate over the flow cells at a flow rate of 40 ul/min in a buffer containing 10 mM HEPES, pH 7.4, 150 mM NaCl, 3.4 mM EDTA, 0.5 mg/ml BSA (bovine serum albumin) and 0.005% (v/v) surfactant P20 at 25 °C. For ALK1-Fc and BMPRII-Fc binding experiments, the surface was regenerated with 4 M Guanidine Hydrochloride while for BMPRII WT and mutant ECD experiments, no regeneration was needed. The kinetic rate constants were obtained by fitting the corrected data to a 1:1 interaction model using Biacore T200 Evaluation Software (version 1.0, GE Healthcare). The equilibrium binding constant *K*_D_ was determined by the ratio of binding rate constants *k*_*d*_*/k*_*a*_.

### Structure of the BMP10:BMPRII complexes

Pro:BMP10 was denatured in 7 M urea solution overnight and the denatured protein was loaded onto a 5 ml HiTrap S column pre-equilibrated in a binding buffer of 20 mM Tris, 25 mM NaCl, 6 M urea, pH 7.4 followed by elution using a NaCl gradient. Fractions containing the denatured BMP10 GF-domain were concentrated to 1 ml followed by a rapid dilution in 19 ml cold refolding buffer (1 M NaCl, 10% glycerol, 3% CHAPS (3-((3-cholamidopropyl) dimethylammonio)-1-propanesulfonate), 2.5% glycine, 5 mM glutamic acid, pH 4.0). Excess of BMPRII ECD was then added in the refolding buffer and left on a roller at 4 °C for 6 days. The refolded mixture was concentrated and further purified using a Superdex 200 16/600 column pre-equilibrated in 20 mM Tris, 150 mM NaCl, pH 7.4. BMP10:BMPRII ECD was concentrated to 4.8 mg/ml for crystallisation trials using the hanging-drop method with 1 μl protein and 1 μl reservoir solution. Crystals were obtained over 4 days at 21 °C in 14% polyethylene glycol (PEG) 3350, 0.19 M ammonium citrate dibasic, 0.02 M sodium citrate tribasic dihydrate, pH 5.8. Crystals were cryo-protected in 30% glycerol in crystallisation reservoir solution and vitrified in liquid nitrogen. Data collection was undertaken at 100 K at Diamond Light Source (DLS, Didcot) on Beamline I04 at a wavelength of 0.91589 Å, and processed in space group *P*2_1_2_1_2_1_ to 1.48 Å using DIALS^35^ and AIMLESS^36^ in the CCP4 suite^37^. The structure was solved by molecular replacement using Phaser^38^, with BMP10 from PDB entry 6SF3 and BMPRII (PDB entry 2HLQ) as the search models. Model building was performed using Coot^39^ and refinement using REFMAC5^40^. The final model was validated using MolProbity^41^. A second crystal form was obtained in 14% PEG 3350, 0.14 M KCl, pH 7.0 in a month and data were collected at I04, DLS at a wavelength of 0.97950 Å. The data were processed in *C*2 space group to 2.40 Å. The structure was determined by molecular replacement in Phaser using the 1.48 Å BMP10:BMPRII structure as a search model. As above, model building was carried out using Coot, refinements using REFMAC5 and phenix.refine^42^ and validation using MolProbity. All the data collection, data reduction, structure determination and refinement statistics are summarised in Supplementary Table 1. The coordinates were deposited in the PDB with accession codes of 7PPA and 7PPB, respectively.

### Structure of the ALK1:BMP10:BMPRII complex

To make the ALK1:BMP10:BMPRII complex, ALK1 was mixed with preformed BMP10:BMPRII complex in a 1.2:1 ratio and the mixture was concentrated to 3.9 mg/ml. Crystallisation was performed by hanging drop method using 1 μl protein and 1 μl reservoir solution. A single crystal cluster was obtained after 15 days at 21 °C in 20% PEG 3350, 0.2 M calcium acetate hydrate, pH 7.5. Crystals were cryo-protected in 30% glycerol in crystallisation reservoir solution and vitrified in liquid nitrogen. Data collection was undertaken at 100 K at Diamond Light Source on Beamline I04-1 at a wavelength of 0.91188 Å and processed in space group *P*2_1_ to 3.60 Å using DIALS and AIMLESS in CCP4 suite. The structure was solved by molecular replacement using Phaser. The ALK1:BMP10 complex from 6SF3 was used as the first search model which gave 4 copies of ALK1:BMP10 1:1 complex in the asymmetric unit forming two copies of ALK1:BMP10 2:2 complex. The BMPRII ECD from the 1.48 Å BMP10:BMPRII structure was used as the second search model and only 1 copy of BMPRII was found by Phaser using the default settings. The second copy of BMPRII was found by searching more deeply in the rotation list, using all peaks above 50% of the top peak in the translation search in Phaser. The last 2 copies were found by using the Brute rotation function generating all orientations within 15 degrees of their expected values (found by superposing the BMP10:BMPRII complex structure on BMP10) and using all these orientations for the following translation search. Since the data were highly anisotropic, anisotropic correction of data used for refinement was performed using the STARANISO Server (Global Phasing Limited, http://staraniso.globalphasing.org/cgi-bin/staraniso.cgi). Model building, refinement and validation were performed as described for the BMP10:BMPRII complexes. All the data collection, data reduction, structure determination and refinement statistics are summarised in Supplementary Table 1. The coordinates were deposited in the PDB with the accession code of 7PPC.

### Structure of the Prodomain-bound BMP10 complexes

Pro:BMP10, with mutations to promote crystal contacts, was concentrated to 5 mg/ml and subjected to a crystal screen at 21 °C with 1.5 μl protein and 1 μl reservoir solution. Crystals (crystal form 1) were obtained in 19% PEG 3350, 0.15 M ammonium tartrate dibasic, 0.02 M sodium cacodylate trihydrate, pH 6.6 after 15 days. Crystals were cryoprotected in 30% glycerol in crystallisation reservoir solution and vitrified in liquid nitrogen. Data collection was undertaken at 100 K at Diamond Light Source on Beamline I04-1 at a wavelength of 0.91589 Å and processed in space group *C*222_1_ to 2.90 Å using DIALS and AIMLESS in CCP4 suite. The structure was solved by molecular replacement using Phaser, with BMP10 from PDB entry 6SF3 and BMP9 prodomain from PDB entry 4YCI as the search models. Model building, refinement and validation were performed as described above.

WT Pro:BMP10 was concentrated to 8.4 mg/ml and crystallised in 20% PEG3350, 0.2 M ammonium tartrate dibasic, pH 6.6 by mixing 1 μl protein and 1 μl reservoir solution at 21 °C for about 100 days (crystal form 2). Data collection was at 100 K at I04-1, DLS at a wavelength of 0.91188 Å, and processed in space group *C*222_1_ to 3.5 Å. The structure was solved by molecular replacement using Phaser, with the crystal form 1 structure as the search model. Model building, refinement and validation were performed as described above for the other structures. All the data collection, data reduction, structure determination and refinement statistics are summarised in Supplementary Table 1. The coordinates were deposited in the PDB with accession codes of 7POI and 7POJ, respectively.

### Structural analysis and sequence alignment

Structural analyses were performed using Coot and Pymol (The PyMOL Molecular Graphics System, Version 2.4.1, Schrödinger, LLC), and figures generated using Pymol. Clustal Omega^43^ was used for sequence alignment. Buried interface area were calculated using QtPISA 2.1.0 in CCP4-7.1.

### Native and SDS-PAGE

Pro:BMP10 was pre-mixed with BMPRII WT or mutant ECDs (at 1:2 and 1:5 molar ratio) in 20 mM Tris, 150 mM NaCl, pH 7.4 in a final volume of 8 ul for 30 minutes at room temperature before fractionation on a 12% native PAGE. After staining with Coomassie Blue, band intensities were quantified by ImageJ (version 1.51 s). To identify the native gel bands, bands were cut out from the native gel and inserted directly into the wells of a 12% gel for SDS-PAGE using a standard protocol.

### Prodomain displacement ELISA

A high binding 96-well plate was coated with 0.25 μg/well of anti-human BMP10 GF-domain antibody (cat. No. MAB2926, R&D Systems) in phosphate-buffered saline (PBS) and incubated in a humidified chamber at 4 °C overnight. The wells were washed with PBS containing 0.05% Tween 20 (PBST) followed by blocking with 1% BSA in PBS (BSA/PBS) at room temperature for 2 hours. In parallel, 25 ng Pro:BMP10 samples were premixed with BMPRII ECD WT (at 1:0, 1:1, 1:5, 1:25, 1:125 molar ratio) or mutants (at 1:125 molar ratio) in 1% BSA/PBS at room temperature for 30 minutes. The plate was washed with PBST before samples were added. After incubation for 2 hours, the plate was washed, and antihuman BMP10 propeptide detection antibody (0.04 μg/well, cat. No. BAF3956, R&D Systems) in 1% BSA/PBS was added. After washing, ExtrAvidin®-Alkaline phosphatase (cat. No. E2636, SIGMA) diluted 1:400 in 1% BSA/PBS was added. The assay was then developed with 0.67 mg/ml 4-Nitrophenyl phosphate disodium salt hexahydrate (cat. No. S0942, SIGMA) in 1 M Diethanolamine, 0.5 mM MgCl_2_, pH 9.8 and absorbance was measured at 405 nm. The experiment was repeated three times, with technical duplicates each time. All values are presented as the ratio of OD_405nm_ of samples to Pro:BMP10 only sample.

### Luciferase reporter assay

HepG2 and 2H-11 cells were seeded in 24-well plates and transfected, respectively, with PEI (Polyethylemimine, Polysciences) and Lipofectamine 3000. BRE-luc transcriptional reporter^44^, β-gal expression plasmid (transfection control) and mutant or WT pcDEF-*BMPR2* plasmids were transfected per well in triplicate. Forty-eight hours post-transfection, the cells were serum-starved overnight before luciferase activity was measured.

### Western blot analyses

Cell lysate was fractionated on 10 or 12% SDS-PAGE and transferred to PVDF membrane. For anti-FLAG blots, after blocking in BSA/PBS, the membrane was incubated with anti-FLAG antibody (cat. No. F1804, Sigma Aldrich, 1:1000 dilution), followed by wash and horseradish peroxidase (HRP)-conjugated secondary antibody (anti-mouse IgG, cat. No. P0447, Dako, 1:2000 dilution). For phosphor-Smad1 blot, an in-house made phosphor-Smad1 antibody (1:1000 dilution) was used which has been validated previously^45^.

### Cell surface biotinylation assay

HEK EBNA cells were seeded in a 24-well plate in duplicate 2 days prior experiment and transfected with 500 ng pcDEF-*BMPR2* plasmid for 24 hours using Lipofectamine™ LTX with PLUS™ Reagent (cat. No. 15338100, Thermo Fisher Scientific). Cells were washed twice with ice-cold PBS (containing Ca^2+^/Mg^2+^) and incubated at 4 °C for 30 min with 0.33 mg/ml Thermo EZ-Link Sulfo-NHS-SS-Biotin in PBS (cat. No. 21328, Thermo Fisher Scientific). Cells were quenched thrice with 50 mM glycine in PBS before lysed on ice for 45 min in 320 μl lysis buffer (100 mM NaCl, 2 mM MgCl_2_, 25 mM Tris-HCl, 1% v/v triton X-100, pH 7.4). Cell lysate was sonicated briefly and centrifuged at 21,000 g for 10 min at 4 °C. Lysate supernatant was loaded (100 μl) in duplicate into 96-well Pierce™ NeutrAvidin™ Coated High Capacity Plates (cat. No. 15507, Thermo Fisher Scientific) and incubated for 2 hours at 4 °C. The plate was blocked with 3% BSA in PBS and incubated with anti-FLAG antibody (F1804, Sigma Aldrich, 1:5000) overnight at 4 °C and then 1 hour at room temperature with anti-mouse HRP secondary antibody (cat. No. P0447, Dako; 1:5000 dilution). Peroxidase activity was detected by incubating with 100 μl/well SIGMAFAST™ OPD (o-Phenylenediamine dihydrochloride) for 30 minutes at room temperature and absorbance was read at 450 nm. Total FLAG-tagged BMPRII in the cell lysate was assessed by immunoblotting.

### RNA extraction and quantitative reverse transcription-PCR (RT-qPCR)

HEK EBNA were seeded at 200,000 cell/well into a 6-well plate followed by 24-hour transfection with 1μg of pcDEF-*BMPR2* plasmid. Total RNA was extracted using RNeasy Mini Kit buffers (Qiagen, West Sussex, UK) and Silica Membrane Mini Spin Columns (EconoSpin) following the manufacturer’s instructions. Equal amounts of RNA (~1 μg) were then reverse transcribed into cDNA using a High Capacity Reverse Transcriptase kit (Applied Biosystems). 2 μl cDNA, 1.8 μl associated premixed primer sets (final concentration = 200 nM), 5 μl 2X SYBR Green JumpStart Taq ReadyMix (Sigma-Aldrich), 0.2 μl ROX reference dye (Invitrogen) and 1 μl DEPC-treated water were prepared into one well of a MicroAmp® Optical 384-Well Reaction Plate (Applied Biosystems) which was then put on a QuantStudio 6 Flex Real-Time PCR System (Applied Biosystems). Amplification reactions were initiated with a 2-minute pre-incubation at 95 °C, followed by 50 amplification cycles of 30-second denaturation at 95 °C, 30 seconds annealing at 55 °C and 30-seconds extension at 72 °C. The following primers were used for the qPCR reactions: human *ID1*: 5′-CTGCTCTACGACATGAACGGC-3′, 5′-TGACGTGCTGGAGAATCTCCA-3′; human β2 microglobulin (*B2M*): 5′-CTCGCGCTACTCTCTCTTTCT-3′, 5′-CATTCTCTGCTGGATGACGTG-3′. The relative expression levels of *ID1* were calculated using the ΔΔCt method by normalizing to *B2M*. Differences in gene expression are presented as the fold change relative to control.

### Statistics

Data analyses were performed using GraphPad Prism 6.0 (GraphPad Software). Results are shown as means ± SEMs. Statistical significance was analysed using One-way ANOVA with Dunnett’s post-test comparing with appropriate controls as indicated in figure legends. Values of *P* < 0.05 were considered significant.

### Reporting summary

Further information on research design is available in the Nature Research Reporting Summary linked to this article.

## Supplementary information

Supplementary Information

Description of Additional Supplementary Files

Supplementary movie 1

Reporting Summary

## Data Availability

The data that support this study are available from the corresponding author upon reasonable request. Coordinates and structure factors for all structures have been deposited to the Protein Data Bank, with the accession numbers of 7PPA, 7PPB, 7PPC, 7POI and 7POJ. Links to the other PDB entries in this paper are: 6SF1, 6SF3, 2HLQ, 4YCI, 4FAO. Source data are provided with this paper.

## Source data

source data
