# Supplementary material for: Crystal structures of BMPRII extracellular domain in binary and ternary receptor complexes with BMP10
Source: Nat Commun. 2022 May 3;13:2395. doi: 10.1038/s41467-022-30111-2 (PMC9064986; doi:10.1038/s41467-022-30111-2)
Supplement: Supplementary file 1 — Supplementary Information [file 41467_2022_30111_MOESM1_ESM.pdf]

SUPPLEMENTARY INFORMATION

Crystal structures of BMPRII extracellular domain in binary and ternary receptor complexes with BMP10

Jingxu Guo[1], Bin Liu[1], Midory Thorikay[2], Minmin Yu[3], Xiaoyan Li[1], Zhen Tong[1], Richard M Salmon[1], Randy J. Read[4], Peter ten Dijke[2], Nicholas W Morrell[1], Wei Li[1,*]

1. Department of Medicine, University of Cambridge School of Clinical Medicine, Cambridge, CB2 0QQ, United Kingdom; 2. Department of Cell and Chemical Biology and Oncode Institute, Leiden University Medical Centre, The Netherlands; 3. MRC Laboratory of Molecular Biology, Francis Crick Avenue, Cambridge CB2 0QH, United Kingdom; 4. Cambridge Institute for Medical Research, The Keith Peters Building, Cambridge Biomedical Campus, Hills Road, Cambridge CB2 0XY, United Kingdom.

**** Corresponding Author:***

Dr Wei Li

Email: wl225@cam.ac.uk

TABLE OF CONTENTS:

Supplementary Table 1-5

1. Supplementary Table 1. Data collection and refinement statistics
2. Supplementary Table 2. Comparison of interactions at the ALK1:BMP10 interface between binary and ternary receptor complexes
3. Supplementary Table 3. List of BMP10:BMPRII 1:1 complexes and their source structures
4. Supplementary Table 4. List of residues with alternative conformation in the 1.48 Å BMP10:BMPRII structure
5. Supplementary Table 5. List of reported BMP9 and BMP10 crystal structures and their protein-protein interaction context

Supplementary Figures 1-14

1. Supplementary Figure 1. Representative electron density of binary and ternary BMPRII receptor complexes
2. Supplementary Figure 2. Different assemblies of the TGF-β family signalling complexes
3. Supplementary Figure 3. Surface plasmon resonance (SPR) experiments investigating the binding affinities of BMP10 for ALK1 in the absence and presence of BMPRII
4. Supplementary Figure 4. Hydrophobic triad in type II receptors, showing on the sequence alignment of those type II receptors with crystal structures of the ligand:receptor complexes
5. Supplementary Figure 5. In support of main Figure 4
6. Supplementary Figure 6. In support of main Figure 5
7. Supplementary Figure 7. Sequence and electron density of Pro:BMP10 structures
8. Supplementary Figure 8. Crystal structures of Pro:BMP10
9. Supplementary Figure 9. Superposition of all BMP9 monomers from different crystal structures
10. Supplementary Figure 10. Control experiments examining the functionality of BMP10 immobilised on a CM5 Biacore chip, in support of Main Figure 7
11. Supplementary Figure 11. Identification of the bands on the native PAGE
12. Supplementary Figure 12. Establishment BMPRII-dependent signalling assays
13. Supplementary Figure 13. Sequence analysis of the ligands for BMPRII–binding features
14. Supplementary Figure 14. *BMPR2* expression data from The Human Protein Atlas

Supplementary References

SUPPLEMENTARY TABLES

Supplementary Table 1. Data collection and refinement statistics (molecular replacement)

	BMP10:BMPRII Crystal form 1 PDB code: 7PPA	BMP10:BMPRII Crystal form 2 PDB code: 7PPB	ALK1:BMP10:BMPRII PDB code: 7PPC	Pro:BMP10 Crystal form 1 PDB code: 7POI	Pro:BMP10 Crystal form 2 PDB code: 7POJ
**Data collection**					
Space group	$P2_12_12_1$	$C2$	$P2_1$	$C222_1$	$C222_1$
Cell dimensions					
a, b, c (Å)	44.7, 47.2, 250.1	119.6, 46.3, 43.8	63.8, 130.4, 88.8	72.9, 214.0, 107.0	143.3, 144.1, 82.1
α, β, γ (°)	90, 90, 90	90, 111, 90	90, 103, 90	90, 90, 90	90, 90, 90
Resolution (Å)	125.04-1.48 (1.51-1.48)	42.82-2.40 (2.49-2.40)	130.39-3.60 (3.94-3.60)$^{\$}$	107.02-2.90 (3.08-2.90)	101.63-3.50 (3.83-3.50)
a*	N/A	N/A	6.90$^{\$\$}$	N/A	N/A
b*	N/A	N/A	3.62$^{\$\$}$	N/A	N/A
c*	N/A	N/A	3.60$^{\$\$}$	N/A	N/A
$CC_{1/2}$	0.999 (0.590)	0.993 (0.745)	0.977 (0.673)	1.000 (0.482)	0.996 (0.521)
R_{sym} or R_{merge}	0.111(1.850)	0.217 (1.048)	0.331 (1.223)	0.166 (3.761)	0.169 (2.702)
I / σI	11.2 (1.4)	6.7 (1.5)	2.5 (0.9)	10.1 (0.5)	6.3 (0.7)
Completeness (%)	100.0 (99.8)	99.0 (96.3)	99.9 (99.5)$^{\$}$	100.0 (100.0)	99.9 (99.5)
At resolution range					
Overall	N/A	N/A	68.2$^{\$\$}$	N/A	N/A
7.67-6.03	N/A	N/A	94.9$^{\$\$}$	N/A	N/A
6.02-4.73	N/A	N/A	72.7$^{\$\$}$	N/A	N/A
4.73-3.60	N/A	N/A	55.0$^{\$\$}$	N/A	N/A
Redundancy	12.7 (11.7)	6.9 (6.1)	4.9 (4.9)	12.9 (12.8)	8.5 (8.5)
**Refinement**					
Resolution (Å)	125.04-1.48	42.82-2.40	86.41-3.60	107.02-2.90	101.63-3.50
No. reflections	89,655	8,799	11,198	18,998	10,978
R_{work} / R_{free}	0.165/0.207	0.206/0.235	0.244/0.283	0.219/0.268	0.275/0.300
No. atoms					
Protein	3,287	1,564	8,136	3,929	3,909
Ligand/ion	6	0	0	38	41
Water	354	38	5	11	1
B-factors (Å^2)					
Protein	36.2	46.5	73.1	112.1	170.1
Ligand/ion	61.4	N/A	N/A	163.7	159.1
Water	38.0	42.4	15.1	83.9	87.6
R.m.s. deviations					
Bond lengths (Å)	0.015	0.002	0.003	0.002	0.002
Bond angles (°)	1.87	0.62	0.62	0.47	0.49
Clash score	3.11	2.61	4.31	3.82	6.27
Ramachandran plot					
Favored (%)	97.2	96.4	94.5	95.2	94.8
Allowed (%)	2.8	3.6	5.5	4.8	5.2
Outliers (%)	0	0	0	0	0

* Reciprocal cell directions

$^{\$}$ Statistics before anisotropy correction.

$^{\$\$}$ Statistics after anisotropy correction.

Supplementary Table 2. Comparison of interactions at the ALK1:BMP10 interface between binary and ternary receptor complexes. The distance further than 4 Å is shown as > 4 Å. Hydrophobic interactions are not listed here. The biggest changes between binary and ternary complexes are shown in bold texts.

	Distance between the ALK1 and BMP10 atoms (Å)							
			ALK1:BMP10		ALK1:BMP10:BMPRII			
	ALK1 residues	BMP10 residues	6SF3	6SF1	Cpx1, chain E	Cpx1, chain F	Cpx2, chain G	Cpx2, chain H
Site I	His40/ND1	Tyr322/OH	3.7	3.6	3.6	n/a[**]	>4	n/a[**]
	Glu58/OE1	Lys386/NZ	3.7	4.0	>4	>4	3.6	n/a[**]
	**Glu59/OE1**	**Ser381/OG**	**2.7**	**2.6**	**n/a**[**]	**>4**	**n/a**[**]	**n/a**[**]
		Ser381/N	3.7	>4	n/a[**]	n/a[**]	n/a[**]	n/a[**]
	Asn71/ND2	Glu362/OE1	3.3	3.2	3.1	3.2	3.6	3.4
	Asn71/N	Glu362/OE1	3.1	3.0	3.2	3.4	3.6	3.3
	Asn71/N	Glu362/OE2	3.0	2.6	3.0	2.8	3.3	3.1
	Leu72/N	Glu362/OE2	2.8	2.7	3.2	3.6	3.7	3.6
Site II	Arg78/NH1 or NH2[*]	Leu402/O	2.9/2.9	2.6/3.0	n/a[**]	3.3/2.5	n/a[**]	2.6/3.8
	Arg78/O	Ser339/OG	2.7	2.7	3.8	2.6	2.8	2.6
	Arg80/N	Gly336/O	2.9	2.7	2.7	2.8	2.7	2.4
	Arg80/N	Ser339/OG	3.5	3.7	>4	3.8	3.2	3.5
	Arg80/NH1[*]	Asp338/OD1 or OD2	2.5/3.2	2.6/3.4	2.9/3.7	2.7/4.0	3.8/3.7	2.6/3.0
	Arg80/NH2[*]	Asp338/OD1	3.5/>4	2.7/3.5	>4/>4	>4/>4	2.9/3.4	>4/>4
	Arg80/NH2	Lys333/O	2.8	3.0	3.9	3.6	3.3	3.0
Site III	His73/NE2	Pro366/O	3.7	3.6	3.2	3.9	3.5	4.0
	Glu75/OE2	Tyr413/OH	3.0	3.2	3.9	3.3	n/a[**]	3.6
Hydrophobic core	**His87/ND1 or NE2**	**Tyr358/OH**	**2.6**	**2.5**	**3.0**	**3.6/3.9**[***]	**>4**	**>4**

[*], those with two numbers are double H-bonds

[**], n/a, not applicable due to poor density for sidechains

[***], His87 in chain F has two conformations, both within H-bind distance to Tyr358/OH.

Supplementary Table 3. List of BMP10:BMPRII 1:1 complexes and their source structures

Complex name	BMP10 chain	BMPRII chain	Source structure
Complex AC	A	C	1.48 Å BMP10:BMPRII
Complex BD	B	D	1.48 Å BMP10:BMPRII
Complex AB	A	B	2.4 Å BMP10:BMPRII
Complex AI	A	I	3.6 Å ALK1:BMP10:BMPRII
Complex BJ	B	J	3.6 Å ALK1:BMP10:BMPRII
Complex CK	C	K	3.6 Å ALK1:BMP10:BMPRII
Complex DL	D	L	3.6 Å ALK1:BMP10:BMPRII

Supplementary Table 4. List of residues with alternative conformation in the 1.48 Å BMP10:BMPRII structure. Those at the BMPRII S107 interaction area are highlighted in red.

ChainA	ChainB	ChainC
K333		Q30
D338		S64
I342		I77
E348		H87
C388	C388	I88
I399		
		V100
		S107
		I108
		S119

Supplementary Table 5. List of reported BMP9 and BMP10 crystal structures and their protein-protein interaction context

	Protein interaction context		PDB code
BMP9	Free form		4MPL
			1ZKZ
			5I05
	Pro:BMP9		4YCG
			4YCI
	Pro:BMP9:ALK1		6SF2
	ENG:BMP9		5HZW
	ALK1:BMP9:ActRIIB		4FAO
BMP10	ALK1:BMP10		6SF3
			6SF1
	BMP10:BMPRII	Complex AB	7PPB*
		Complex BD	7PPA*
		Complex AC	7PPA*
	ALK1:BMP10:BMPRII	Complex AI	7PPC*
		Complex BJ	7PPC*
		Complex CK	7PPC*
		Complex DL	7PPC*
	Pro:BMP10 crystal form 1		7POI*
	Pro:BMP10 crystal form 2		7POJ*

*, crystal structures reported in this paper.

SUPPLEMENTARY FIGURES

Supplementary Figure 1

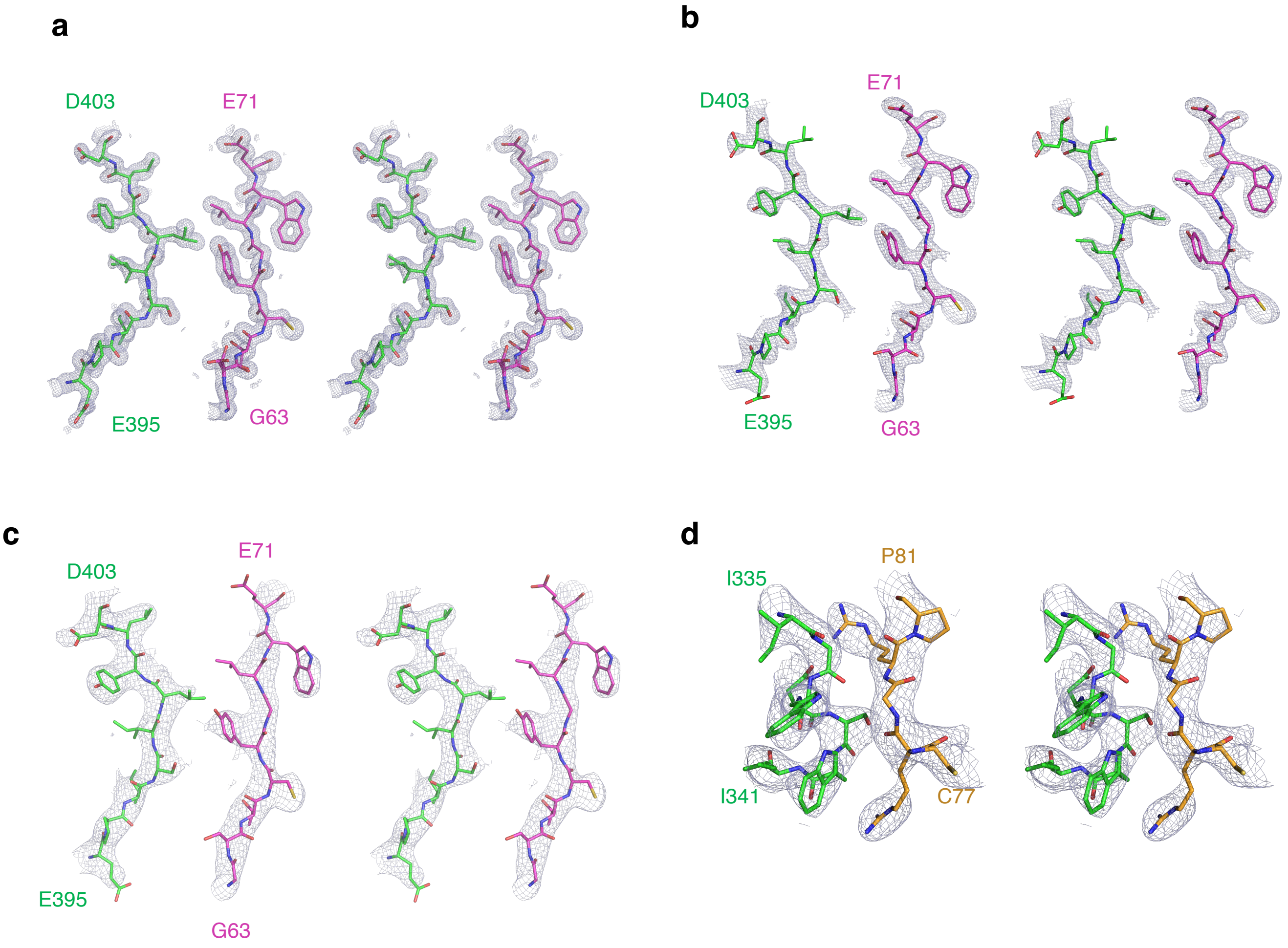

Supplementary Figure 1. **Representative electron density of binary and ternary BMPRII receptor complexes**. The 2Fo-Fc electron density (contoured at 1.5$\boldsymbol{\sigma}$) overlaid with the final refined model. BMP10 is coloured in green, BMPRII coloured in magenta, ALK1 coloured yellow. **a.** 1.48 Å BMP10:BMPRII complex; **b**. 2.4 Å BMP10:BMPRII complex; **c&d**. ALK1:BMP10:BMPRII complex.

Supplementary Figure 2

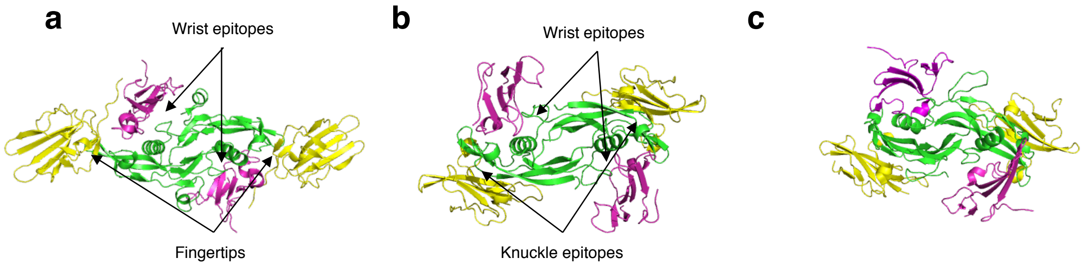

Supplementary Figure 2. Different assemblies of the TGF-β family signalling complexes. Ligands are shown in green, type I receptors in magenta, type II receptors in yellow. TGF-β family ligands are mostly homodimers, with each monomer being typically described as a hand, and the receptor binding sites designated as wrist epitopes, knuckle epitopes and fingertips. **a**. TGF-β subfamily signalling complex represented by ALK5:TGF-β3:TGFβRII structure (PDB code: 2PJY)[1]. **b**. BMP subfamily signalling complex with ActRIIA/B represented by ALK1:BMP9:ActRIIB structure (PDB code: 4FAO)[2]. **c**. Activin subfamily signalling complex represented by ALK5:GDF11:ActRIIB structure (PDB code: 6MAC)[3].

Supplementary Figure 3

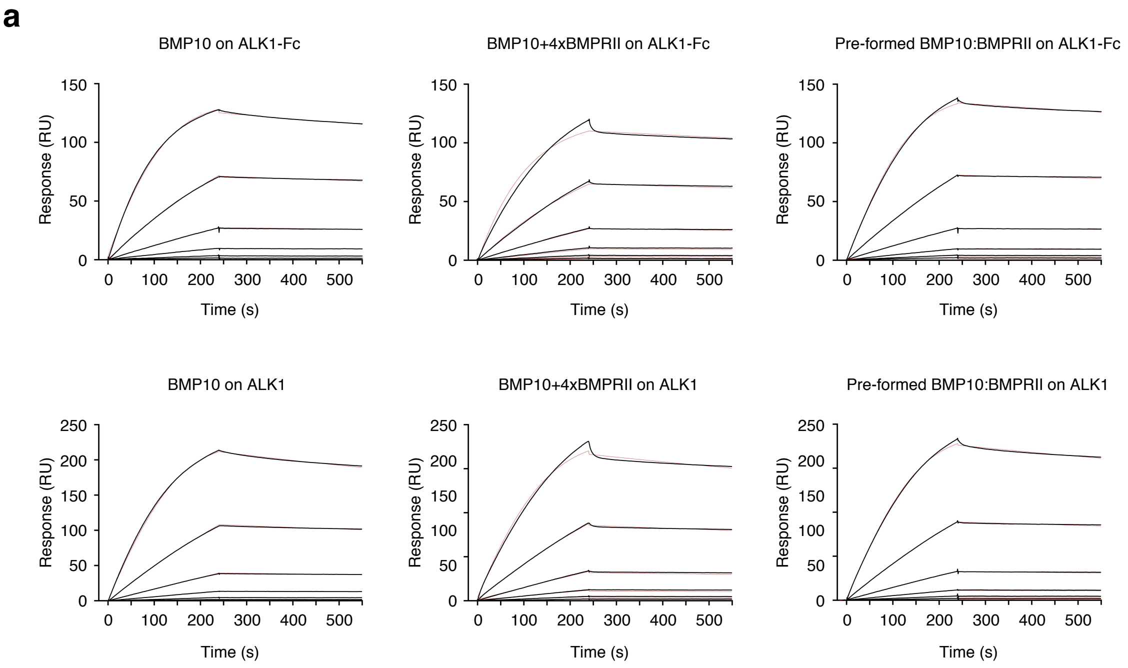

b

	ALK1-Fc			ALK1		
	*k*a ($M^{-1}s^{-1}$)	*k*d (s^{-1})	*K*D (M)	*k*a ($M^{-1}s^{-1}$)	*k*d (s^{-1})	*K*D (M)
**BMP10**	1.15E+06	2.82E-04	2.53E-10	1.33E+06	4.17E-04	3.27E-10
**BMP10+4xBMPRII**	6.88E+05	2.74E-04	4.05E-10	9.03E+05	3.57E-04	3.96E-10
**BMP10:BMPRII**	9.36E+05	2.43E-04	2.67E-10	1.12E+06	3.46E-04	3.09E-10

Supplementary Figure 3. Surface plasmon resonance (SPR) experiments investigating the binding affinities of BMP10 for ALK1 in the absence and presence of BMPRII. a. SPR sensorgrams of BMP10, BMP10+4xBMPRII, or pre-formed BMP10:BMPRII complex binding to ALK1 or ALK1-Fc immobilised on a CM5 sensor chip. For BMP10+4xBMPRII sample, BMP10 was pre-mixed with BMPRII with a molar ratio of 1:4 (monomer) at room temperature for 30 minutes before being flowed on the chip. Pre-formed BMP10:BMPRII complex was the same binary complex protein stock used for crystallisation. **b.** Summary of kinetic parameters from the SPR assays. s, second; M, Molar.

Supplementary Figure 4

```
                                                              β1        α1
BMPRII:BMP10_C            27 ---------------------SQNQERLCAFKDDPYQQDLGIGESR-----ISHENG 56
ActRIIA_M:BMP2(2GOO)      20 ------------------AILGRSETQECLFFNANWEKD-----------RTNQTG 46
ActRIIA_M:BMP7(1LX5)      20 ------------------AILGRSETQECLFFNANWEKD-----------RTNQTG 46
ActRIIB:BMP9(4FAO)        19 ------------------SGRGEAETRECIYYNANWELE-----------RTNQSG 45
ActRIIB:BMP2(2H62)        19 ------------------SGRGEAETRECIYYNANWELE-----------RTNQSG 45

ActRIIB_M:GDF11(6MAC)     19 ------------------SGRGEAETRECIYYNANWELE-----------RTNQSG 45
ActRIIB_M:ActA(1NYS)      19 ------------------SGRGEAETRECIYYNANWELE-----------RTNQSG 45
ActRIIB_M:ActA(1S4Y)      19 ------------------SGRGEAETRECIYYNANWELE-----------RTNQSG 45

AMHRII:AMH(7L0J)          18 ----------------------PPNRRTCVFFEAPGVRGSTKTLGELLDTGTELPR 51

TGFβRII:TGF-β1(3KFD)      23 TIPPHVQKSVNNDMIVTDNNGAVKFPQLCKFCDVRFSTCDNQKSC-----MSNCSI 73
TGFβRII:TGF-β3(2PJY,1KTZ) 23 TIPPHVQKSVNNDMIVTDNNGAVKFPQLCKFCDVRFSTCDNQKSC-----MSNCSI 73
                                                                  Finger 1 loop

                               β2             β3         β4                 α2
BMPRII:BMP10_C            57 TILCSK----GSTCYGLWEKSKGDINLVKQGCWSHIGDP-----QECH-YEECVVT 102
ActRIIA_M:BMP2(2GOO)      47 VEPCYGDKDKRRHCFATWKNISGSIEIVKQGCWLDD--------INCYDRTDCVEK 94
ActRIIA_M:BMP7(1LX5)      47 VEPCYGDKDKRRHCFATWKNISGSIEIVKQGCWLDD--------INCYDRTDCVEK 94
ActRIIB:BMP9(4FAO)        46 LERCEGEQDKRLHCYASWRNSSGTIELVKKGCWLDD--------FNCYDRQECVAT 93
ActRIIB:BMP2(2H62)        46 LERCEGEQDKRLHCYASWRNSSGTIELVKKGCWLDD--------FNCYDRQECVAT 93

ActRIIB_M:GDF11(6MAC)     46 LERCEGEQDKRLHCYASWRNSSGTIELVKKGCWLDD--------FNCYDRQECVAT 93
ActRIIB_M:ActA(1NYS)      46 LERCEGEQDKRLHCYASWRNSSGTIELVKKGCWLDD--------FNCYDRQECVAT 93
ActRIIB_M:ActA(1S4Y)      46 LERCEGEQDKRLHCYASWRNSSGTIELVKKGCWLDD--------FNCYDRQECVAT 93

AMHRII:AMH(7L0J)          52 AIRCLY----SRCCFGIWNLTQDRAQVEMQGCRDSD-------EPGCE-SLHCDPS 95

TGFβRII:TGF-β1(3KFD)      74 TSICEKP---QEVCVAVWRKNDENITL-ETVCHDPKLPYHDFILEDA-ASPKCIMK 124
TGFβRII:TGF-β3(2PJY,1KTZ) 74 TSICEKP---QEVCVAVWRKNDENITL-ETVCHDPKLPYHDFILEDA-ASPKCIMK 124
                             Finger 1 loop                            A-loop

                                         β5
BMPRII:BMP10_C           103 TTPPSIQNGTYRFCCCSTDLCNVNFTENFPP----PDTTPLSPPHSFNRDET--   150
ActRIIA_M:BMP2(2GOO)      95 KDSP-----EVYFCCCEGNMCNEKFSYFPEMEVTQPTSNPVTPKPP--------   135
ActRIIA_M:BMP7(1LX5)      95 KDSP-----EVYFCCCEGNMCNEKFSYFPEMEVTQPTSNPVTPKPP--------   135
ActRIIB:BMP9(4FAO)        94 EENP-----QVYFCCCEGNFCNERFTHLPEAGGPEVTYEPPPTAPTLLT-----   137
ActRIIB:BMP2(2H62)        94 EENP-----QVYFCCCEGNFCNERFTHLPEAGGPEVTYEPPPTAPTLLT-----   137

ActRIIB_M:GDF11(6MAC)     94 EENP-----QVYFCCCEGNFCNERFTHLPEPGGPEVTYEPPPTAPTLLT-----   137
ActRIIB_M:ActA(1NYS)      94 EENP-----QVYFCCCEGNFCNERFTHLPEPGGPEVTYEPPPTAPTLLT-----   137
ActRIIB_M:ActA(1S4Y)      94 EENP-----QVYFCCCEGNFCNERFTHLPEPGGPEVTYEPPPTAPTLLT-----   137

AMHRII:AMH(7L0J)          96 PRAHPSPGSTLFTCSCGTDFCNANYSHLPPPGSPGTPGSQGPQAAPGESIWMAL   149

TGFβRII:TGF-β1(3KFD)     125 EKKKP--GETFFMCSCSSDECNDNIIFSEEYNTSNPDLLLVIFQ----------   166
TGFβRII:TGF-β3(2PJY,1KTZ)125 EKKKP--GETFFMCSCSSDECNDNIIFSEEYNTSNPDLLLVIFQ----------   166
```

Supplementary Figure 4. Hydrophobic triad in type II receptors, showing on the sequence alignment of those type II receptors with crystal structures of the ligand:receptor complexes. The names of the sequences are the crystal structures with pdb access codes in brackets. The 'hydrophobic triad' is indicated by the three red boxes. Residues at the ligand interaction interface are coloured in blue. Secondary structure elements are marked over the sequence according to chain C in the 1.48Å BMP10:BMPRII structure, except for α1-helix which is marked according to the 2HLQ BMPRII structure due to the lack of electron density in the BMP10:BMPRII structure. The finger 1 loop and the A-loop in TGFβRII are marked under the sequences. Residues that are not visible in chain C of the 1.48 Å BMP10:BMPRII structure due to lack of electron density are coloured in grey. Sequences from mouse origin are labelled with '_M' in their names, and the residues which are different in their human homologs are marked by the black boxes.

Supplementary Figure 5

a

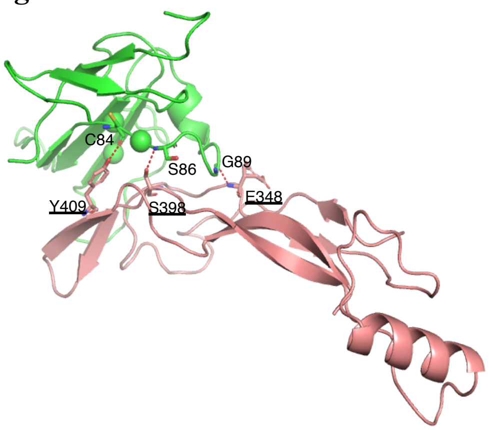

b

	Buried surface area upon complex formation (Å2)								
	On BMP9	On ActRIIB	Total	On BMP9	On ActRIIB	Total	On BMP9	On ActRIIB	Total
Chain IDs	A	E		B	F		H	L	
	865.2	899.1	1764.3	844.0	871.9	1715.9	801.5	837.7	1639.2
Chain IDs	G	K		N	R		M	Q	
	793.6	818.4	1612.0	768.3	789.8	1558.1	888.6	904.5	1793.1
Chain IDs	b	f		a	e		T	X	
	814.4	844.6	1659.0	808.0	847.8	1655.8	761.8	790.2	1552.0
Chain IDs	S	W		h	l		g	k	
	828.8	839.0	1667.8	812.3	842.0	1654.3	787.8	824.9	1612.7

c

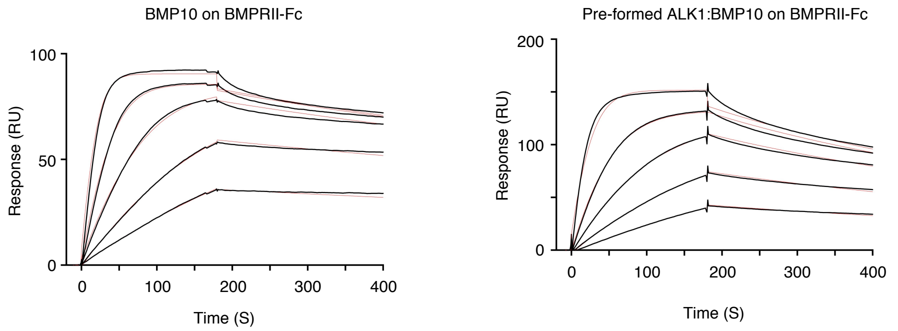

	BMPRII-Fc		
	k_a ($M^{-1}s^{-1}$)	k_d (s^{-1})	K_D (M)
**BMP10**	2.79E+06	8.38E-04	3.38E-10
**ALK1:BMP10**	1.15E+06	1.38E-03	1.60E-09

Supplementary Figure 5. In support of main Figure 4. a. The conserved interactions from BMPRII C84, S86 and G89 contact three out of four β-strand fingers in BMP10. Complex AC is shown, with BMP10 coloured in coral and BMPRII in green. **b**. Buried surface area between BMP9 and ActRIIB in ALK1:BMP9:ActRIIB complexes (PDB: 4FAO). There are 12 ternary complexes in an asymmetric unit in 4FAO. Buried surface areas between each BMP9:ActRIIB pair, calculated using QtPISA 2.1.0 in CCP4 (version 7.1), are shown,. **c.** SPR sensorgrams of BMP10 and pre-formed ALK1:BMP10 complex binding to BMPRII-Fc immobilised on a CM5 chip. The kinetic parameters are shown below. The measured affinity of BMP10 for BMPRII-Fc is comparable with those reported in the literature[2,6]. ALK1:BMP10 complex was formed by incubating ALK1 ECD with BMP10 with a molar ratio of 2:1 (monomer) at room temperature for 30 minutes.

Supplementary Figure 6

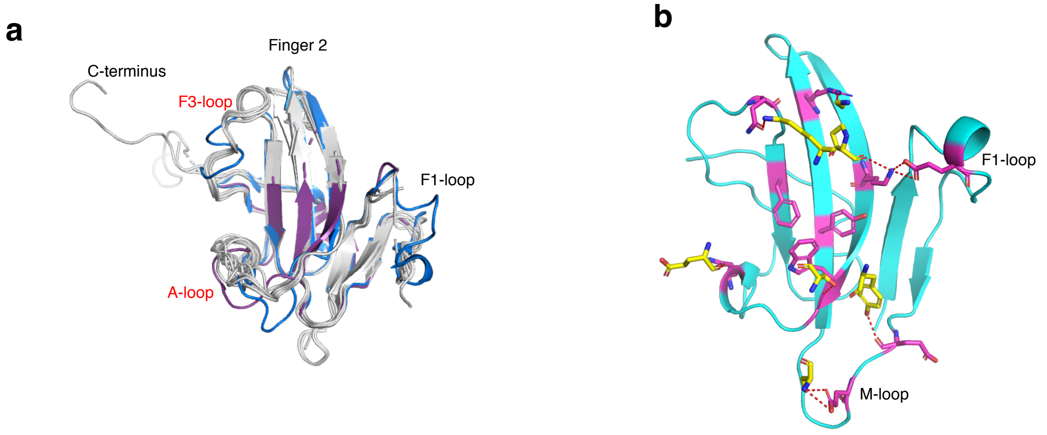

Supplementary Figure 6. In support of main Figure 5. a. Comparison of BMPRII structures in the ligand bound and unbound forms. Apart from the F1-loop and the C-terminus, seven BMP10-bound BMPRII structures (all shown in grey) are almost identical in the A-loop and the F3-loop. Small variations can be seen in finger 2 conformation. When superimposed with free BMPRII structures (2HLR in dark purple (1.2 Å), 2HLQ in dark blue (1.45 Å)), significant shifts can be seen in the F1-loop, the A-loop and the F3-loop. **b**. BMP9 interactions with ActRIIB (PDB code: 4FAO). ActRIIB (in cyan cartoon) with interaction residues shown in magenta sticks. BMP9 residues that make H-bond interactions are shown in yellow sticks. Red dashes denote H-bonds.

Supplementary Figure 7

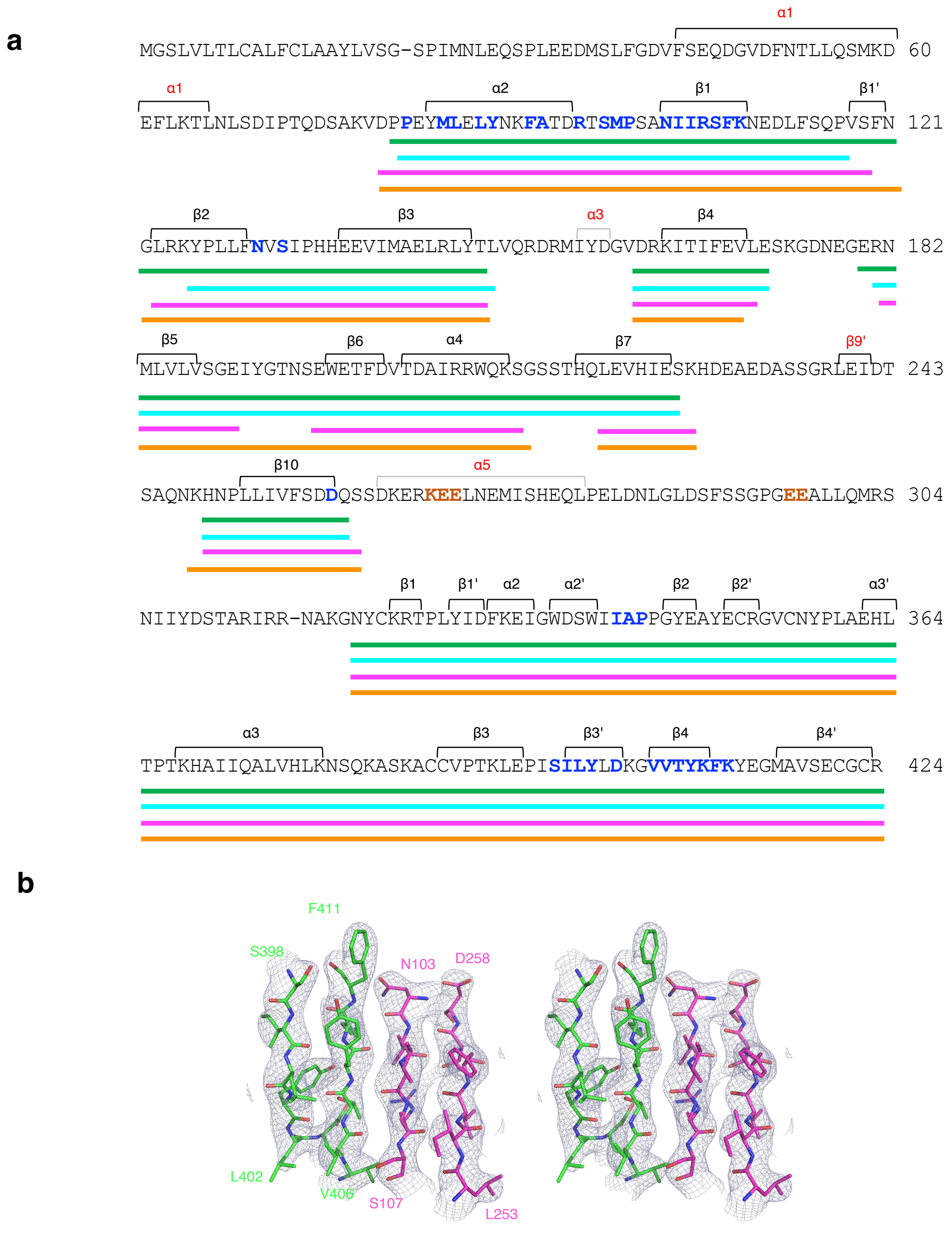

Supplementary Figure 7. Sequence and electron density of Pro:BMP10 structures. **a.** Sequence coverage in Pro:BMP10 crystal structures shown as lines below the sequence, crystal form 1 in green and cyan, and crystal form 2 in magenta and orange. Residues at the prodomain:GF-domain interface are in bold and coloured in blue. Mutations in the prodomain in crystal form 1 for promoting crystal contacts are shown in bold and coloured in dark orange. Secondary structure elements are marked over the sequence according to Pro:BMP10 structure (black) or according to Mi *et al* [4] (red). **b**. A stereo image of the 2Fo-Fc map (contoured at 1.5 δ) from the pro (magenta) – GF-domain (green) interface of the Pro:BMP10 crystal form 1.

Supplementary Figure 8

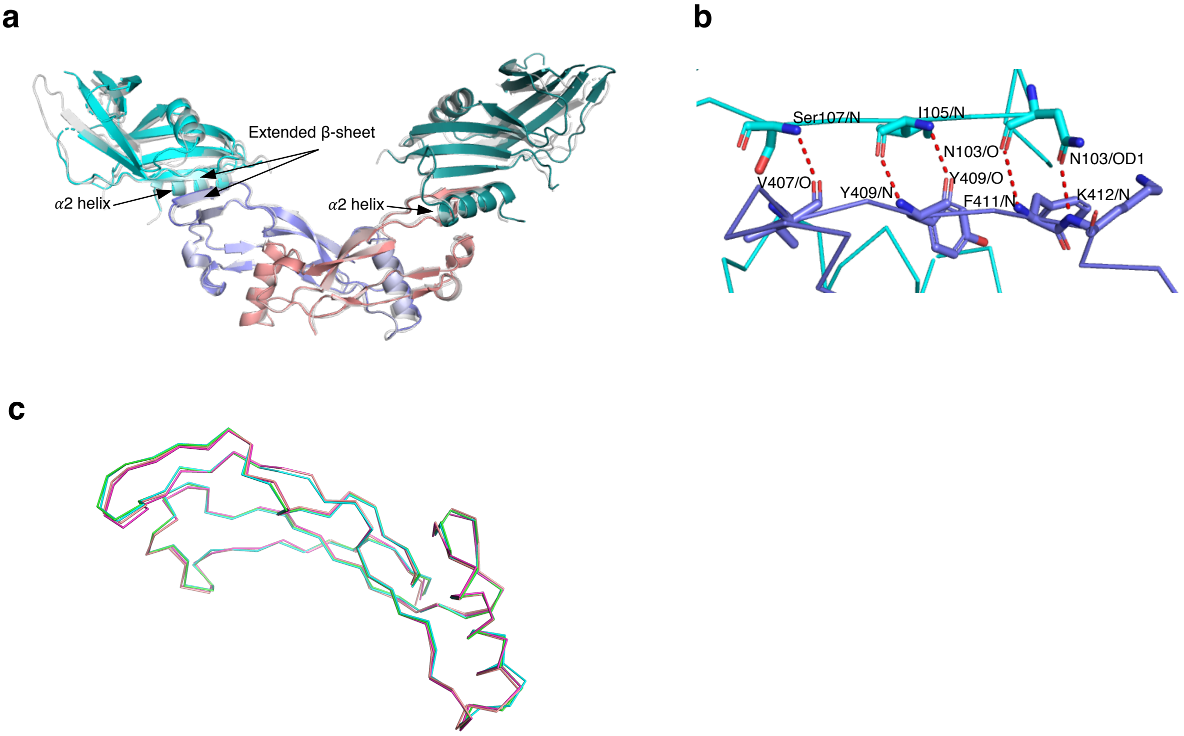

Supplementary Figure 8. **Crystal structures of Pro:BMP10**. **a**. Overlay of the two Pro:BMP10 structures. Crystal form 1 is coloured in purple and orange for BMP10, dark green and cyan for prodomain. Crystal form 2 is shown in grey and semi-transparent for all chains. **b.** A zoomed-in view of the extended β-sheet interactions between the GF-domain (purple) and the prodomain (cyan). Crystal form 1 which has a higher resolution is used for this analysis. **c**. Overlay of four BMP10 monomers from the two Pro:BMP10 complexes shown as ribbons. Chains coloured in green and cyan are from crystal form 1, in magenta and orange are from crystal form 2.

Supplementary Figure 9

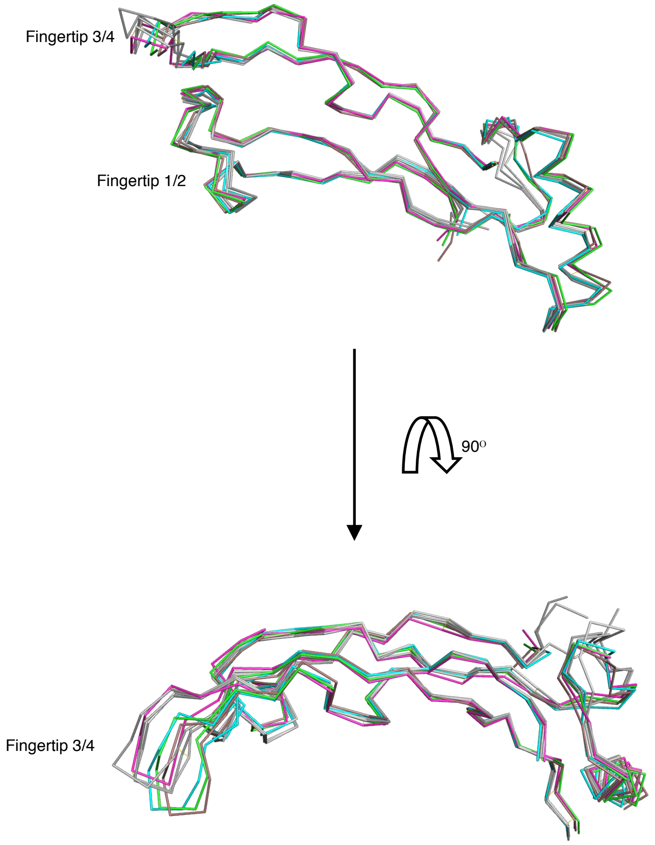

Supplementary Figure 9. Superposition of BMP9 monomers from different crystal structures. All the structures and PDB codes are listed in Supplementary Table 5. Coloured in grey are from free BMP9 structures (PDB codes: 4MPL, 1ZKZ, 5I05), in magenta from ALK1:BMP9:ActRIIB complex (PDB code: 4FAO), in cyan from BMP9 in complex with ENG orphan domain (PDB code: 5HZW), in green from BMP9 in complex with prodomain and ALK1 (PDB code: 6SF2), and in brown from prodomain bound BMP9 (PDB code: 4YCG). A zoomed-in view of the low panel fingertip 3/4 is shown in Fig 6c with the same colour scheme.

Supplementary Figure 10

a

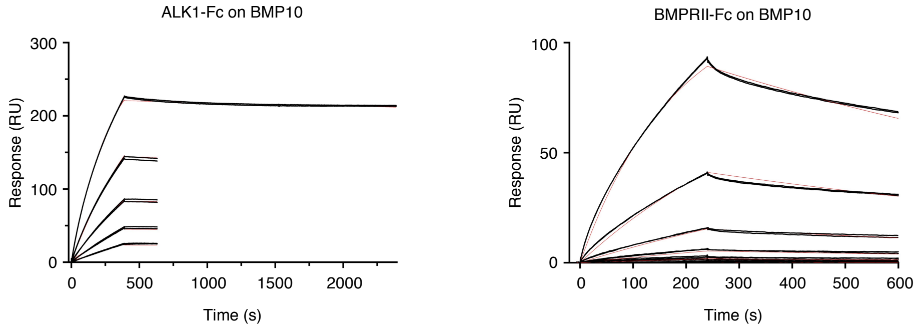

b

	BMP10		
	k_a ($M^{-1}s^{-1}$)	k_d (s^{-1})	K_D (M)
**ALK1-Fc**	4.70E+04	2.94E-05	6.24E-10
**BMPRII-Fc**	1.92E+05	8,57E-04	4.47E-09

Supplementary Figure 10. Control experiments examining the functionality of BMP10 immobilised on a CM5 Biacore chip, in support of Main Figure 7. a. SPR sensorgrams of ALK1-Fc and BMPRII-Fc binding to BMP10. **b.** Summary of kinetic parameters from the SPR assay. As shown here and in Main Figure 7a, BMP10 immobilised on the SPR chip can interact with both ALK1 and BMPRII, and as expected[2], its affinity for ALK1-Fc is nearly 10-fold higher than for BMPRII-Fc. Of note, both ALK1-Fc and BMPRII-Fc showed around 20-fold lower affinity compared with those measured by BMP10 flowing on ALK1-Fc or BMPRII-Fc immobilised sensor chips[2,5,6], probably due to the limitation of BMP10 orientations when immobilised on the CM5 chip. Binding to BMPRII ECD monomer (Main Fig 7 a&f) is about 266-fold weaker than BMPRII-Fc. This is likely due to the BMPRII-Fc being a dimer because the difference is due to the off-rate rather than the on-rate. Overall, the control experiments support that this BMP10 chip provides a functional tool for evaluating the effects of BMPRII mutations on BMP10-binding affinity.

Supplementary Figure 11

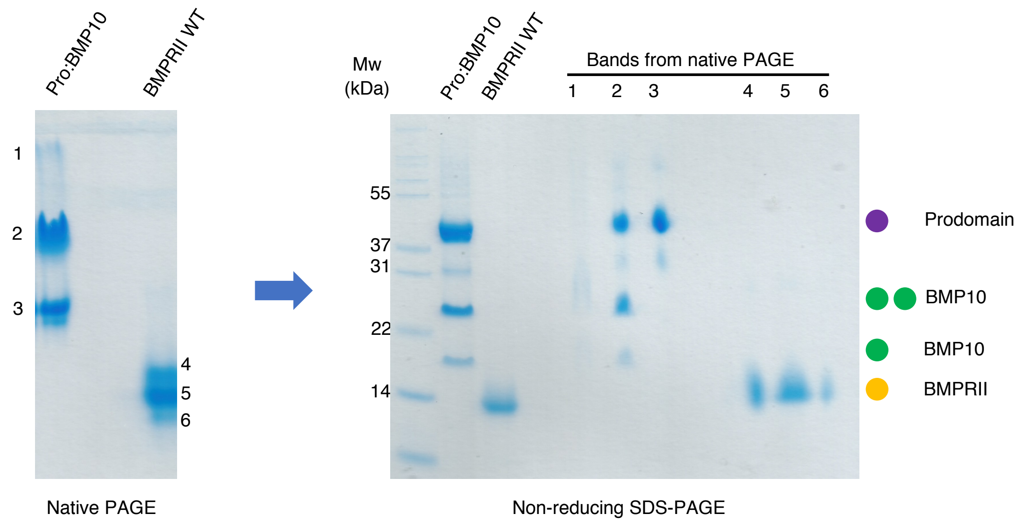

Supplementary Figure 11. Identification of the bands on the native PAGE. *Left*, Native PAGE for Pro:BMP10 and BMPRII WT. *Right*, Bands 1 to 6 on the native PAGE were cut out and re-run on a 12% non-reducing SDS-PAGE, with Pro:BMP10 and BMPRII WT as controls, to confirm their identity. Experiments were performed three times with similar result obtained. Coloured circles are used to label different proteins, with a single-circle indicating a monomer and double-circles indicating a dimer. The key to the coloured circles are shown. WT = wild type, Pro:BMP10 = non-covalent complex of BMP10 prodomain with its growth factor domain.

Supplementary Figure 12

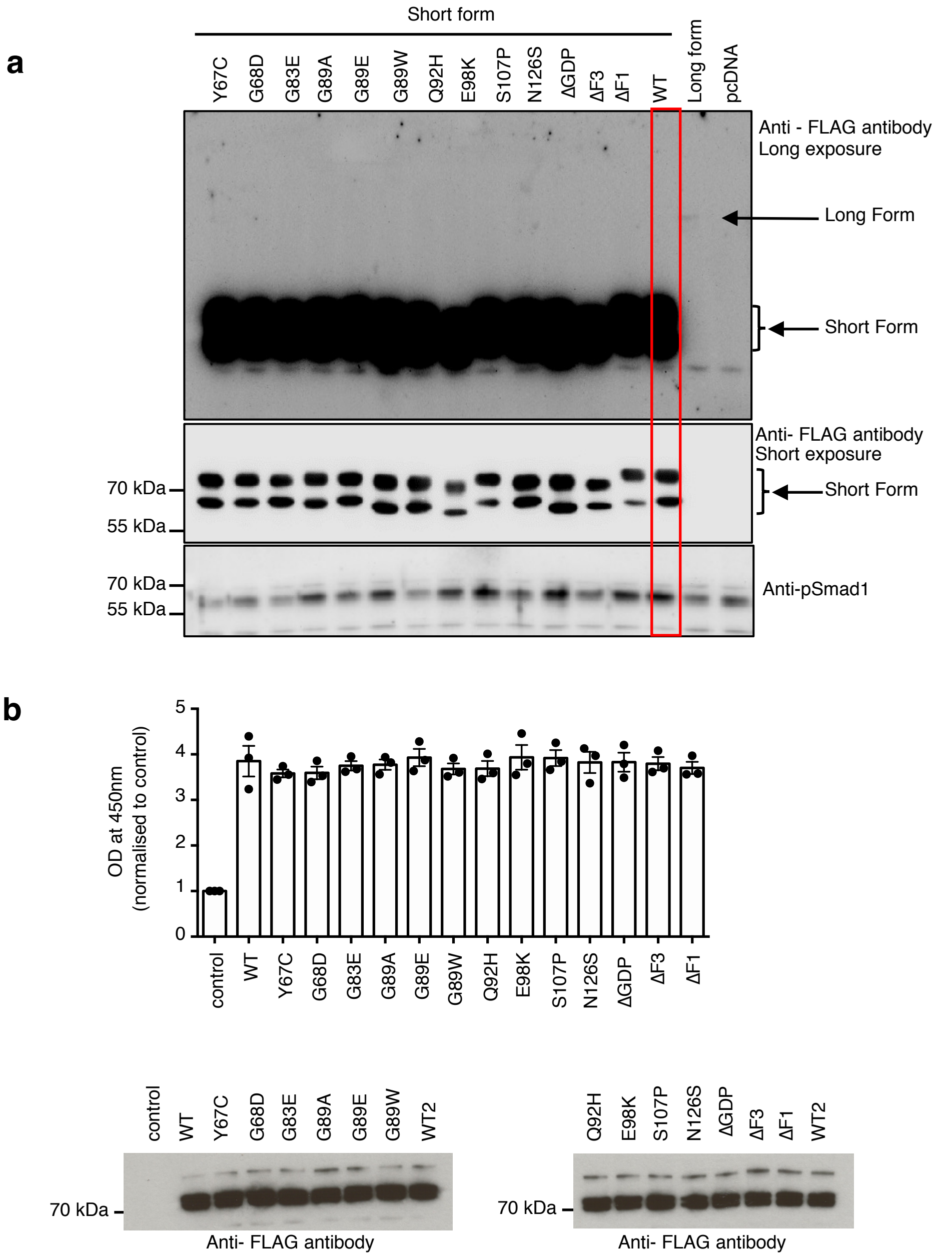

Supplementary Figure 12. Establishment of BMPRII-dependent signalling assays. **a**. Transfection in HEK293 cells, western blots detecting transfection efficiency using anti-FLAG antibodies, and the effect of FLAG-tagged BMPRII overexpression on Smad1 phosphorylation. Red box highlights the transfection result of the wild type (WT) short form BMPRII. Experiments were performed three times with similar results obtained. **b**. Surface biotinylation experiment. HEK EBNA cells were transfected with FLAG-tagged BMPRII short form expression plasmids, containing the mutations as indicated. *Upper panel*: surface biotinylation-ELISA result. Briefly, cells were subject to surface biotinylation as described in the Materials and Methods. Subsequently, equal volume of cell lysate were captured on a NeutrAvidin plate, followed by anti-FLAG and HRP-conjugated secondary antibody detection. Three independent experiments were performed. Data are normalised to vector only transfection control. Data are presented as mean values +/- SEM. *Lower panel*, Equal amount of total cell lysates were subject to immunoblot analysis using anti-FLAG antibody to monitor the total FLAG-tagged BMPRII across different transfections. WT2 is a WT control sample, loaded in the same amount on both gels to allow comparison. The experiment was performed twice, and the result of one representative experiment is shown. Source data are provided as a Source Data file.

Supplementary Figure 13

```
                                                          Contacting F3-loop in BMPRII

BMP10   -------------------------------NAKGNYCKRTPLYIDFK-EIGWDSWIIAP 344
ActB    ---------------------------GLECDGRTNLCCRQQFFIDFR-LIGWNDWIIAP 324
Nodal   ----------------------------HHLPDRSQLCRKVKFQVDFN-LIGWGSWIIYP 268
BMP9    ------------------------------SAGAGSHCQKTSLRVNFE-DIGWDSWIIAP 348
ActA    ---------------------------GLECDGKVNICCKKQFFVSFK-DIGWNDWIIAP 342
BMP4    ----------------------SPKHHSQRARKKNKNCRRHSLYVDFS-DVGWNDWIVAP 329
BMP6    SASSRRRQQSRNRSTQSQDVARVSSASDYNSSELKTACRKHELYVSFQ-DLGWQDWIIAP 433
BMP7    STGSKQRSQNRSKTPKNQEALRMANVAENSSSDQRQACKKHELYVSFR-DLGWQDWIIAP 351
GDF11   -----------------------NLGLDCDEHSSESRCCRYPLTVDFE-AFGW-DWIIAP 333
TGF-β1  -----------------------ALDTNYCFSSTEKNCCVRQLYIDFRKDLGW-KWIHEP 314
                                             *        *    **  **  *

BMP10   PGYEAYECRGVCNYPLAEHLTPT----KHAIIQALVHLKNSQKASKACCVPTKLEPISIL 400
ActB    TGYYGNYCEGSCPAYLAGVPGSASSFHTAVVNQYRMRGLNPG-TVNSCCIPTKLSTMSML 383
Nodal   KQYNAYRCEGECPNPVGEEFHPT----NHAYIQSLLKRYQPHRVPSTCCAPVKTKPLSML 324
BMP9    KEYEAYECKGGCFFPLADDVTPT----KHAIVQTLVHLKFPTKVGKACCVPTKLSPISVL 404
ActA    SGYHANYCEGECPSHIAGTSGSSLSFHSTVINHYRMRGHSPFANLKSCCVPTKLRPMSML 402
BMP4    PGYQAFYCHGDCPFPLADHLNST----NHAIVQTLVNSVNS-SIPKACCVPTELSAISML 384
BMP6    KGYAANYCDGECSFPLNAHMNAT----NHAIVQTLVHLMNPEYVPKPCCAPTKLNAISVL 489
BMP7    EGYAAYYCEGECAFPLNSYMNAT----NHAIVQTLVHFINPETVPKPCCAPTQLNAISVL 407
GDF11   KRYKANYCSGQCEYMFMQKYPHT----------HLVQQANPRGSAGPCCTPTKMSPINML 383
TGF-β1  KGYHANFCLGPCPYIWSLDTQYS-------KVLALYNQHNPGASAAPCCVPQALEPLPIV 367
          *    * * *                                   ** *

               ↓ ↓
BMP10   YLD-KGVVTYKFKYEGMAVSECGCR 424
ActB    YFDDEYNIVKR-DVPNMIVEECGCA 407
Nodal   YVDN-GRVLLD-HHKDMIVEECGCL 347
BMP9    YKDDMGVPTLKYHYEGMSVAECGCR 429
ActA    YYDDGQNIIKK-DIQNMIVEECGCS 426
BMP4    YLDEYDKVVLK-NYQEMVVEGCGCR 408
BMP6    YFDDNSNVILK-KYRNMVVRACGCH 513
BMP7    YFDDSSNVILK-KYRNMVVRACGCH 431
GDF11   YFNDKQQIIYG-KIPGMVVDRCGCS 407
TGF-β1  YYVGRKP-KVE-QLSNMIVRSCKCS 390
        *               * *  * *
```

Supplementary Figure 13. Sequence analysis of the ligands for BMPRII–binding features. Sequence alignment of those ligands that have shown to bind BMPRII-Fc with different affinities[6], as well as TGF-β1 which does not bind to BMPRII as a negative control. * marks residues that are conserved across all ligands. Coloured residues are those making direct interactions with BMPRII in BMP10:BMPRII structures. Magenta: sidechain interactions; Red: mainchain interactions; Light brown: at the hydrophobic core. V407 and Y409 (indicated with arrows) also contribute to the hydrophobic interaction. Residues in other ligands that are identical to BMPRII-binding residues in BMP10 are highlighted with the same colour scheme as in BMP10. Ligands are arranged according to the measured affinities for BMPRII[6], from top to bottom, tightest to weakest, specifically, BMP10, ActB and Nodal: 0.2 – 0.8 nM; BMP9 and ActA, 7.4 – 9.0 nM; BMP6, BMP7 and GDF11, 39.0 – 53.0 nM. Sequence alignment was performed using Clustal Omega[7].

Supplementary Figure 14

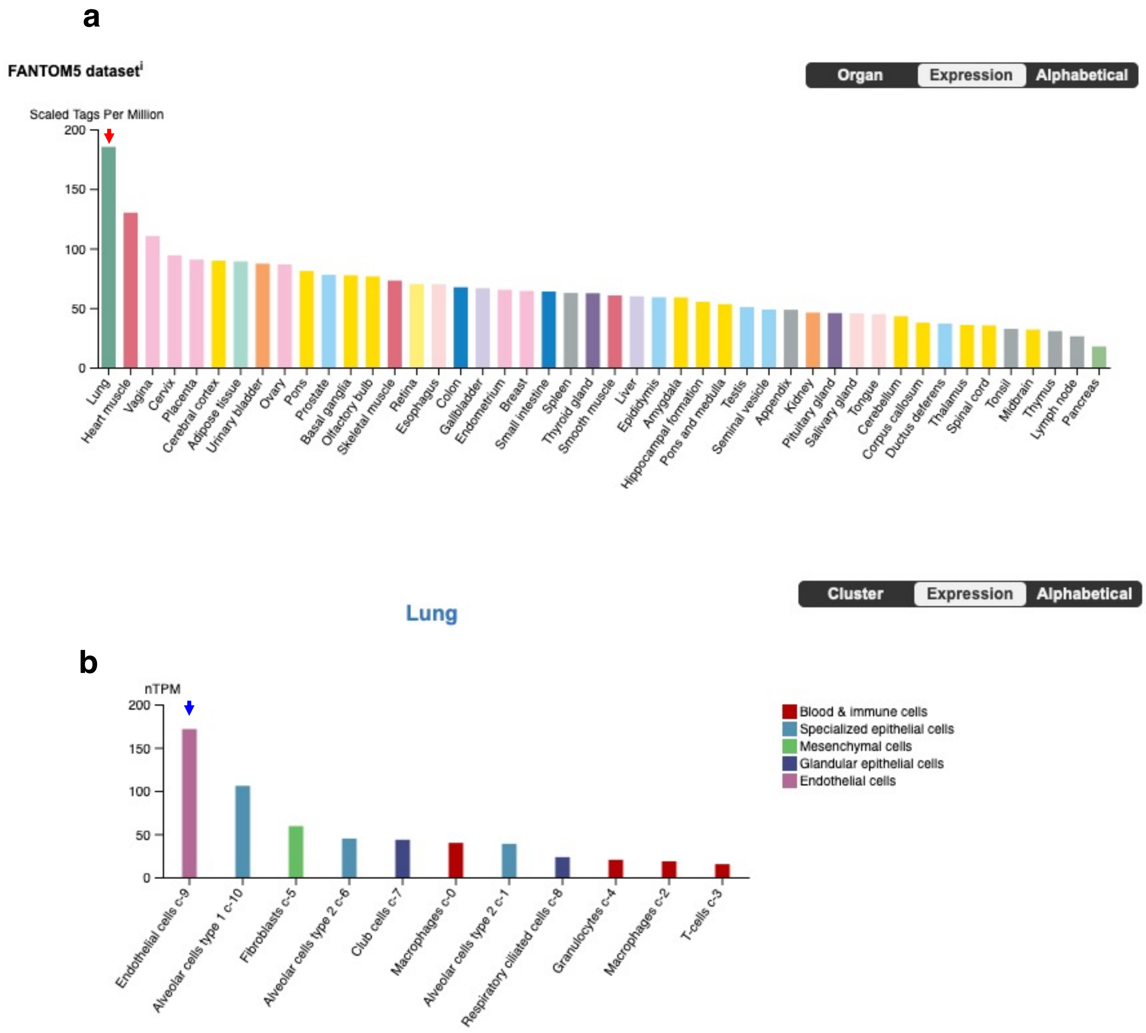

Supplementary Figure 14. *BMPR2* expression data from The Human Protein Atlas. a, Lung has the highest *BMPR2* RNA expression (indicated by the red arrow) among the tissues according to FANTOM5 dataset[8] https://v21.proteinatlas.org/ENSG00000204217-BMPR2/tissue. **b**, Endothelial cells have the highest *BMPR2* RNA expression (indicated by the blue arrow) among the lung single cell types https://v21.proteinatlas.org/ENSG00000204217-BMPR2/celltype.

SUPPLEMENTARY REFERENCES

1. Groppe, J. et al. Cooperative assembly of TGF-beta superfamily signaling complexes is mediated by two disparate mechanisms and distinct modes of receptor binding. Mol. Cell 29, 157-168, doi:10.1016/j.molcel.2007.11.039 (2008).
2. Townson, S. A. et al. Specificity and structure of a high affinity activin receptor-like kinase 1 (ALK1) signaling complex. J. Biol. Chem. 287, 27313-27325, doi:10.1074/jbc.M112.377960 (2012).
3. Goebel, E. J. et al. Structural characterization of an activin class ternary receptor complex reveals a third paradigm for receptor specificity. Proc. Natl. Acad. Sci. U. S. A. 116, 15505-15513, doi:10.1073/pnas.1906253116 (2019).
4. Mi, L. Z. et al. Structure of bone morphogenetic protein 9 procomplex. Proc. Natl. Acad. Sci. U. S. A. 112, 3710-3715, doi:10.1073/pnas.1501303112 (2015).
5. Salmon, R. M. *et al.* Molecular basis of ALK1-mediated signalling by BMP9/BMP10 and their prodomain-bound forms. *Nat Commun* **11**, 1621, doi:10.1038/s41467-020-15425-3 (2020).
6. Aykul, S. & Martinez-Hackert, E. Transforming Growth Factor-beta Family Ligands Can Function as Antagonists by Competing for Type II Receptor Binding. *J. Biol. Chem.* **291**, 10792-10804, doi:10.1074/jbc.M115.713487 (2016).
7. Sievers, F. et al. Fast, scalable generation of high-quality protein multiple sequence alignments using Clustal Omega. Mol. Syst. Biol. 7, 539, (2011).
8. Lizio M, et al. Gateways to the FANTOM5 promoter level mammalian expression atlas. Genome Biol 16: 22 (2015). 10.1186/s13059-014-0560-6
