# Supplementary material for: Crystal structures of BMPRII extracellular domain in binary and ternary receptor complexes with BMP10
Source: Nat Commun. 2022 May 3;13:2395. doi: 10.1038/s41467-022-30111-2 (PMC9064986; doi:10.1038/s41467-022-30111-2)
Supplement: Supplementary file 2 — Description of Additional Supplementary Files [file 41467_2022_30111_MOESM2_ESM.docx]

**Supplementary Movie 1: Conformational flexibility in BMP10:BMPRII 1:1 complex.** Movie showing the movement from complex BD to complex CK. BMP10 is shown in coral, BMPRII in green. BMP10 hinge residue F411 and BMRPII docking residue G89 are shown in spheres. The Hydrogen-bond interactions between BMP10 Y409 and E348 to BMPRII C84 and G89, respectively, are shown in dashed red lines.
