# Supplementary material for: Crystal structures of BMPRII extracellular domain in binary and ternary receptor complexes with BMP10
Source: Nat Commun. 2022 May 3;13:2395. doi: 10.1038/s41467-022-30111-2 (PMC9064986; doi:10.1038/s41467-022-30111-2)
Supplement: Supplementary file 4 — Reporting Summary [file 41467_2022_30111_MOESM4_ESM.pdf]

nature portfolio

Corresponding author(s): Wei Li

Last updated by author(s): Mar 14, 2022

Reporting Summary

Nature Portfolio wishes to improve the reproducibility of the work that we publish. This form provides structure for consistency and transparency in reporting. For further information on Nature Portfolio policies, see our Editorial Policies and the Editorial Policy Checklist.

Statistics

For all statistical analyses, confirm that the following items are present in the figure legend, table legend, main text, or Methods section.

n/a	Confirmed	
☐	☒	The exact sample size (*n*) for each experimental group/condition, given as a discrete number and unit of measurement
☐	☒	A statement on whether measurements were taken from distinct samples or whether the same sample was measured repeatedly
☐	☒	The statistical test(s) used AND whether they are one- or two-sided<br>*Only common tests should be described solely by name; describe more complex techniques in the Methods section.*
☒	☐	A description of all covariates tested
☒	☐	A description of any assumptions or corrections, such as tests of normality and adjustment for multiple comparisons
☐	☒	A full description of the statistical parameters including central tendency (e.g. means) or other basic estimates (e.g. regression coefficient) AND variation (e.g. standard deviation) or associated estimates of uncertainty (e.g. confidence intervals)
☐	☒	For null hypothesis testing, the test statistic (e.g. *F*, *t*, *r*) with confidence intervals, effect sizes, degrees of freedom and *P* value noted<br>*Give P values as exact values whenever suitable.*
☒	☐	For Bayesian analysis, information on the choice of priors and Markov chain Monte Carlo settings
☒	☐	For hierarchical and complex designs, identification of the appropriate level for tests and full reporting of outcomes
☒	☐	Estimates of effect sizes (e.g. Cohen's *d*, Pearson's *r*), indicating how they were calculated

Our web collection on statistics for biologists contains articles on many of the points above.

Software and code

Policy information about availability of computer code

Data collection	All X-ray diffraction data were collected at Diamond Light Source. Methods section contains detailed information of all the softwares used in the data collection. All softwares are commercial or open source. The versions of the softwares used in this study are: Biacore T200 Evaluation Software (version 1.0, GE Healthcare), Crystallography softwares: AutoProc (version 1.0.5), DIALS (version 1.14.5 for 7PPA and 7PPC, version 1.14.13 for 7PPC), AIMLESS (version 0.7.4), CCP4 (version 7.0 for 7PPA, 7PPC, 7POI and 7POJ, version 7.1 for 7PPB), Phaser (version 2.8.3), Coot (version 0.9), REFMAC (version 5.8.0267), Phenix.refine and Molprobity in PHENIX package (version 1.17_3644 for 7POJ, version 1.19.2_4158 for 7PPB, 7PPC, 7POI), STARANISO Sever (Global Phasing Limited, https://staraniso.globalphasing.org/cgi-bin/staraniso.cgi)
Data analysis	Methods section contains detailed information of all the softwares used in the data analysis. All softwares are commercial or open source, including Pymol (The PyMOL Molecular Graphics System, Version 2.4.1, Schrödinger, LLC), QtPISA (version 2.1.0), Clustal Omega (https://www.ebi.ac.uk/Tools/msa/clustalo/), ImageJ (NIH, Java 1.8.0_171), GraphPad Prism 6.0 (GraphPad Software),

For manuscripts utilizing custom algorithms or software that are central to the research but not yet described in published literature, software must be made available to editors and reviewers. We strongly encourage code deposition in a community repository (e.g. GitHub). See the Nature Portfolio guidelines for submitting code & software for further information.

March 2021

Data

Policy information about availability of data

All manuscripts must include a data availability statement. This statement should provide the following information, where applicable:

- Accession codes, unique identifiers, or web links for publicly available datasets
- A description of any restrictions on data availability
- For clinical datasets or third party data, please ensure that the statement adheres to our policy

Coordinates and structure factors for all structures have been deposited to the Protein Data Bank, with the accession numbers of 7PPA, 7PPB, 7PPC, 7POI and 7POJ. Links to the other PDB entries in this paper are: 6SF1 [http://doi.org/10.2210/pdb6sf1/pdb], 6SF3 [http://doi.org/10.2210/pdb6sf3/pdb], 2HLQ [http://doi.org/10.2210/pdb2hlq/pdb], 4YCI [http://doi.org/10.2210/pdb4yci/pdb], 4FAO [http://doi.org/10.2210/pdb4fao/pdb].

Field-specific reporting

Please select the one below that is the best fit for your research. If you are not sure, read the appropriate sections before making your selection.

[x] Life sciences [ ] Behavioural & social sciences [ ] Ecological, evolutionary & environmental sciences

For a reference copy of the document with all sections, see nature.com/documents/nr-reporting-summary-flat.pdf

Life sciences study design

All studies must disclose on these points even when the disclosure is negative.

Sample size	No sample-size calculations were performed. Sample size was determined to be adequate based on the magnitude and consistency of measurable differences between groups. For all signalling assays, we typically perform 3-5 independent experiments to allow statistical analysis and robust conclusions to be drawn. For in vitro prodomain displacement experiment, two different experimental approaches (Fig 8b and Fig 8d) were employed. For each approach, 3-5 independent experiments were performed to allow statistical analysis and robust conclusions to be drawn. For those experiments that are not quantitative, at least two repeats were performed to confirm the observation.
Data exclusions	No data exclusions in this manuscript.
Replication	Replicate experiments were successful. For quantitative measurements, three or more independent experiments were carried out and statistical analysis performed. For non-quantitative biochemical assays, each experimental condition was repeated at least one more time to confirm the observation. X-ray structural coordinates were validated by MolProbity server before deposition to Protein Data Bank and independent validation reports obtained from Protein Data Bank.
Randomization	No randomization was required in this manuscript. Randomization is not relevant in the cell signalling assays because all the cells were under identical conditions before different treatment samples applied.
Blinding	The SPR binding dataset and the cell signalling datasets were performed by different investigators in a blinded manner. Fig 8b native gel assay and quantification were performed by different investigators in a blinded manner. Fig 8d data collection and analysis were performed by different investigators in a blinded manner.

Reporting for specific materials, systems and methods

We require information from authors about some types of materials, experimental systems and methods used in many studies. Here, indicate whether each material, system or method listed is relevant to your study. If you are not sure if a list item applies to your research, read the appropriate section before selecting a response.

Materials & experimental systems

n/a	Involved in the study	
[ ]	[x]	Antibodies
[ ]	[x]	Eukaryotic cell lines
[x]	[ ]	Palaeontology and archaeology
[x]	[ ]	Animals and other organisms
[x]	[ ]	Human research participants
[x]	[ ]	Clinical data
[x]	[ ]	Dual use research of concern

Methods

n/a	Involved in the study	
[x]	[ ]	ChIP-seq
[x]	[ ]	Flow cytometry
[x]	[ ]	MRI-based neuroimaging

Antibodies

Antibodies used	Antibodies used for pro-BMP10 ELISA are: MAB2926 (monoclocal mouse IgG2A, Clone #462732) and BAF3956 from R&D Systems.

	Anti-FLAG antibody (F1804, clone M2) was purchased from Sigma Aldrich. PhosphoSmad1 antibody was generated in house which has been validated in reference PMID: 9738456. Horseradish peroxidase (HRP)-conjugated anti-mouse IgG was from Dako (Cat. No. _0447). The dilution factors or the actual quantities of antibody used in each assay are provided in the Methods.
Validation	The antibody pair for pro-BMP10 ELISA has been validated in PMID: 31661308. Anti-FLAG antibody has been validated by the vendor for immunoblotting, immunoprecipitation, immunohistochemistry, immunofluorescence and immunocytochemistry, optimized for single banded detection of FLAG fusion proteins in mammalian, plants and bacterial expression systems. Anti-phosphoSmad1 antibody has been validated in reference PMID: 9738456.

Eukaryotic cell lines

Policy information about cell lines

Cell line source(s)	Hep-G2 cells (cat. No. HB-8065), 2H-11 cells (cat. No. CRL-2163) and HEK EBNA cells (cat. No. CRL-10852) were all purchased from ATCC.
Authentication	All cells used in this study have been authenticated by the vendor ATCC. All human cell lines (HepG2 and HEK) were authenticated by STR profiling (to confirm the identity and purity). Mouse 2H-11 endothelial cell lines was characterized for expression of endothelial markers (PMID: 33644053). All cell lines used in his study were frequently tested for absence of mycoplasma. All cell lines used in experiments were not kept in culture for more than 2 months. All cell lines were checked to maintain similar morphology in cell culture.
Mycoplasma contamination	All cells tested negative for mycoplasma
Commonly misidentified lines (See ICLAC register)	No cell lines used are listed in the database of commonly misidentified cell lines.
